# Controlled Synthesis of Carbon-Supported Pt-Based Electrocatalysts for Proton Exchange Membrane Fuel Cells

**DOI:** 10.1007/s41918-022-00173-3

**Published:** 2022-09-24

**Authors:** Huiyuan Liu, Jian Zhao, Xianguo Li

**Affiliations:** Department of Mechanical and Mechatronics Engineering, University of Waterloo, 200 University Avenue West, Waterloo, ON N2L 3G1 Canada

**Keywords:** Carbon-supported Pt-based electrocatalysts, Synthesis, Shape, Functionalization of commercial carbon support, Postsynthesis treatment

## Abstract

Proton exchange membrane fuel cells are playing an increasing role in postpandemic economic recovery and climate action plans. However, their performance, cost, and durability are significantly related to Pt-based electrocatalysts, hampering their large-scale commercial application. Hence, considerable efforts have been devoted to improving the activity and durability of Pt-based electrocatalysts by controlled synthesis in recent years as an effective method for decreasing Pt use, and consequently, the cost. Therefore, this review article focuses on the synthesis processes of carbon-supported Pt-based electrocatalysts, which significantly affect the nanoparticle size, shape, and dispersion on supports and thus the activity and durability of the prepared electrocatalysts. The reviewed processes include (i) the functionalization of a commercial carbon support for enhanced catalyst–support interaction and additional catalytic effects, (ii) the methods for loading Pt-based electrocatalysts onto a carbon support that impact the manufacturing costs of electrocatalysts, (iii) the preparation of spherical and nonspherical Pt-based electrocatalysts (polyhedrons, nanocages, nanoframes, one- and two-dimensional nanostructures), and (iv) the postsynthesis treatments of supported electrocatalysts. The influences of the supports, key experimental parameters, and postsynthesis treatments on Pt-based electrocatalysts are scrutinized in detail. Future research directions are outlined, including (i) the full exploitation of the potential functionalization of commercial carbon supports, (ii) scaled-up one-pot synthesis of carbon-supported Pt-based electrocatalysts, and (iii) simplification of postsynthesis treatments. One-pot synthesis in aqueous instead of organic reaction systems and the minimal use of organic ligands are preferred to simplify the synthesis and postsynthesis treatment processes and to promote the mass production of commercial carbon-supported Pt-based electrocatalysts.

**Graphical Abstract:**

This review focuses on the synthesis process of Pt-based electrocatalysts/C to develop aqueous one-pot synthesis at large-scale production for PEMFC stack application.

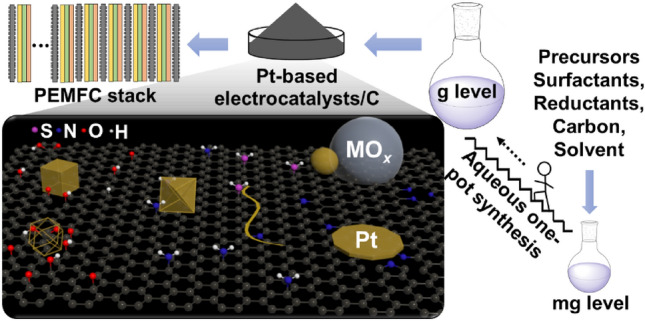

## Introduction

After several decades of intensive research, development, and demonstration, proton exchange membrane fuel cells (PEMFCs) have reached the early stage of commercial deployment due to their high energy efficiency, potential for zero-emission lifecycles, and low-temperature operation [1–4]. Recently, the major economies of the world have released their respective national or regional hydrogen strategies as a part of postpandemic economic recovery and climate action plans, and PEMFCs are expected to play a significant role. However, the widespread commercial application of PEMFCs is still facing technical challenges because of their performance, cost, and durability, which are significantly related to their electrocatalysts. Hence, considerable efforts have been devoted to improving the activity and durability of electrocatalysts as an effective method to reduce the use of noble metal electrocatalysts and, consequently, the cost of PEMFCs.

In PEMFCs, the chemical energy stored in hydrogen and oxygen is directly converted to electrical energy through electrochemical reactions with hydrogen oxidized at the anode and oxygen reduced at the cathode. Because PEMFCs operate with acidic electrolytes at low temperatures, they require electrocatalysts with high activity and durability to accelerate electrochemical processes, especially the cathodic oxygen reduction reaction (ORR), which is a multielectron and multistep reaction with sluggish kinetics, in the oxidizing, acidic and high-potential environment at the cathode. Currently, carbon-supported Pt-based electrocatalysts have been considered to have the highest catalytic activity and durability for the ORR among available electrocatalysts [5, 6]. However, the high cost and the insufficient activity and stability associated with the present carbon-supported Pt-based electrocatalysts hinder the widespread practical application of PEMFCs. To reduce the use of Pt and thus the cost, three major technical pathways have been widely employed. The first pathway is to prepare active and stable carbon-supported Pt-based electrocatalysts that can potentially be used for PEMFCs at a commercial level [7, 8]. The second pathway is to optimize the microstructure of the catalyst layer and promote the formation of a three-phase boundary, enhancing Pt utilization and thus decreasing the use of Pt. This pathway can be achieved by optimizing the composition, formulation, and dispersion process of electrocatalyst ink and the catalyst layer fabrication process [9], as well as exploring a structure-ordered catalyst layer [10–12]; notably, the recent progress of these methods has been well reviewed [9, 13, 14]. The third pathway is to develop nonprecious metal-based electrocatalysts with high active site density and durability [15, 16], as reviewed in Refs. [17–19]. However, although the performance of nonprecious metal-based electrocatalysts has recently been greatly improved, there is still a long way to go before their practical substitution of Pt-based electrocatalysts [19]. Therefore, the present review is focused on the first pathway.

**Fig. 1:**
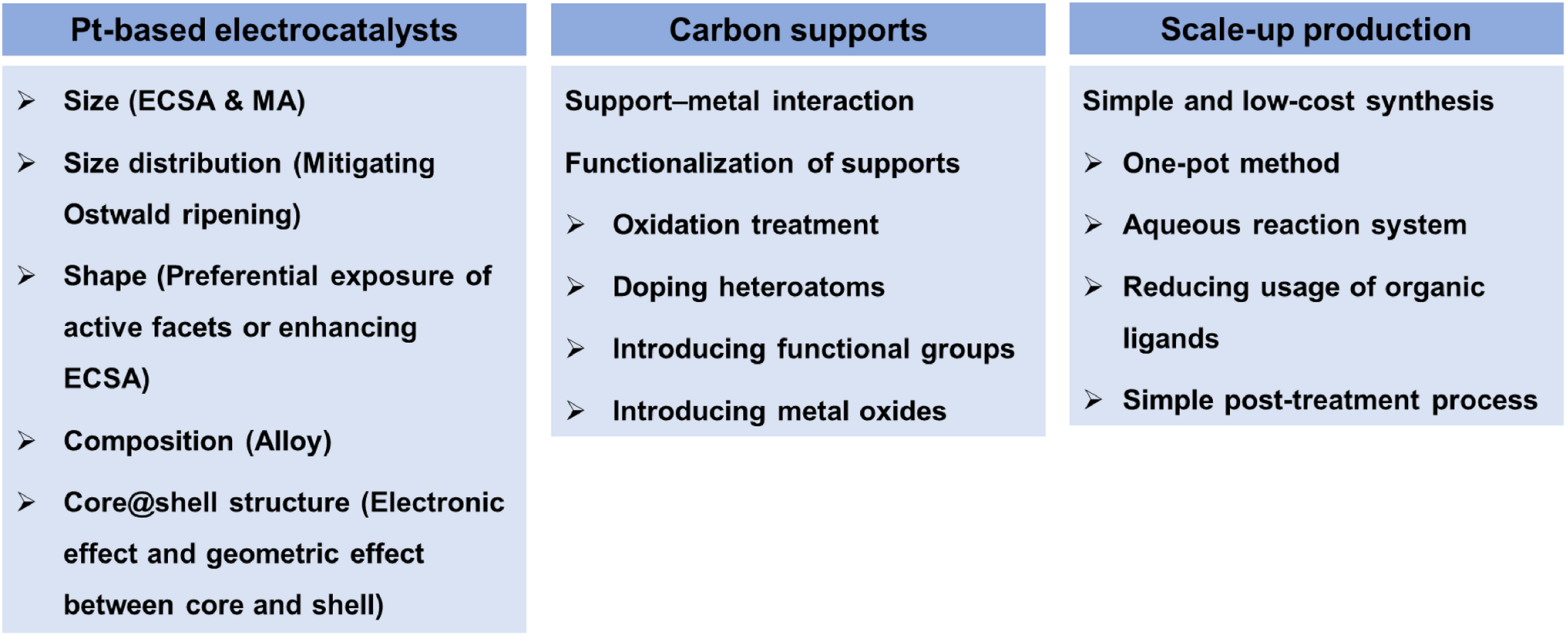
Main approaches to reducing the costs related to Pt-based electrocatalysts (ECSA: electrochemical active surface area; MA: mass activity)

To tackle the remaining technical challenges, significant efforts have been made, and progress has been achieved by designing and preparing novel Pt-based electrocatalysts [20–22]. As shown in Fig. 1, these studies include a wide range of considerations.

1. *The size control of Pt-based particles* [23–27]. Electrocatalytic reactions occur on the electrocatalyst surface, which increases significantly when the electrocatalyst particle size is decreased for a given amount of the electrocatalyst. In other words, the electrochemical active surface area (ECSA) increases with a decrease in the particle size of the catalyst particle [23]. However, the specific activity (the activity per unit surface area of Pt nanoparticles) commonly decreases with a decrease in the particle size due to the increased proportion of Pt atoms located at the edges, vertices, or defect sites, which are regarded as less active sites than crystal facets [24, 26]. Therefore, the mass activity (the catalytic activity per unit mass of Pt) has a maximum value at a certain Pt size, that is, approximately 2–3 nm [23, 24, 26]. To improve Pt utilization, single-atom Pt-based electrocatalysts have been actively investigated; however, it is still challenging to increase the density of active sites, i.e., the number of single-atom Pt atoms on a support, and to stabilize single-atom Pt during PEMFC operation [28, 29]. Therefore, single-atom electrocatalysts are still not commercially available for PEMFC applications and are excluded from this review.
2. *The particle size distribution of Pt-based electrocatalysts* [30]. A narrow size distribution can mitigate the degradation of the Pt-based electrocatalyst caused by Ostwald ripening, thus enhancing the stability and durability of Pt-based electrocatalysts.
3. *The composition of Pt-based electrocatalysts* [31–33]. Alloying Pt with one or more transition metals can modulate the electronic properties of Pt, and subsequently, the electrocatalytic activity by changing the coordination environment or modifying the electronic environment of Pt.
4. *The support* [6, 34]. Since the Pt particle is small, often several nanometers, it is often loaded on support materials to improve the dispersion of Pt particles. The support not only acts as a support but also has a vital influence on the activity and durability of supported Pt-based electrocatalysts due to the existence of metal–support interactions. Therefore, to boost the performance of Pt-based electrocatalysts, supports need to be functionalized to adjust the metal–support interactions.
5. *The shape of Pt-based electrocatalysts* [35, 36]. Highly reactive crystal facets can be selectively displayed by controlling the shape of Pt-based electrocatalysts, significantly enhancing their electrocatalytic activity. Several specific shapes have shown to be beneficial to improve the activity and durability of electrocatalysts. Nanocages or nanoframes simultaneously expose external and interior surfaces, increasing the ECSA. One- or two-dimensional (1D or 2D) nanostructures have more contact area between Pt-based electrocatalysts and supports than nanoparticles, thus improving the durability.
6. *The core/shell structure* [20, 37, 38]. Since only Pt atoms on the electrocatalyst surface contribute to the activity, Pt loading can be considerably reduced by using Pt or Pt alloys as the shell, and other metals, alloys or nonmetals as the core. This structure can also improve the activity of electrocatalysts by controlling the electronic and geometric effects between the core and shell.
7. *Simple low-cost synthesis of carbon-supported Pt-based electrocatalysts* [8, 22, 39]. The cost of carbon-supported Pt-based electrocatalysts will be effectively reduced if the complex multistep fabrication process can be simplified, which is also favorable for scaling up the production of Pt-based electrocatalysts.

In recent decades, a large quantity of Pt-based electrocatalysts with various controlled sizes, size distributions, compositions, shapes, and/or structures have been successfully synthesized in various reaction systems, and the activities and durabilities of these electrocatalysts have been significantly improved [20, 40, 41]. Notably, PtCo alloy electrocatalysts have been practically applied in some cases (e.g., Toyota Mirai [42]), and its development has been well summarized in a recent review [43]. In addition, to summarize the development of Pt-based electrocatalysts, there have been many reviews focusing on the relation between the size, composition, shape, structure, or support on the activity and durability of Pt-based electrocatalysts [29, 44–59]. However, only a few [60, 61] have focused on the relation between the experimental details of the synthesis process and the prepared Pt-based electrocatalysts. These details include the reactant mixture composition including the selection of precursors [62, 63], reductants [64, 65], solvents [66], and structure-capping agents [67] that are used; the reaction conditions including the reaction temperature [68] and temperature ramp rate [69]; and the impacts of these experimental parameters on the prepared Pt-based electrocatalyst, which is very important to control particle size and shape as well as develop feasible reaction systems for mass production.

Herein, the present review focuses on the current development of the controlled synthesis of Pt-based electrocatalysts for PEMFCs; for instance, controlling the abovementioned experimental parameters that affect the size, size distribution, carbon support functionalization, shape and simple low-cost synthesis of these electrocatalysts. Here, less attention is given to the development of how to acquire Pt-based alloys and core/shell structures, which have been well reviewed in the literature [47, 60, 70]. First, an overview of carbon supports and the effects of the structure and surface properties of commercial carbon materials on Pt-based electrocatalysts are presented. Then, the methods for loading Pt-based electrocatalysts onto carbon by one-pot synthesis and ex situ mixing methods, and experimental studies on the size and shape of Pt-based electrocatalysts, particularly strategies to control the size and shape of Pt-based electrocatalysts, are summarized by analyzing recent experimental studies of Pt-based electrocatalysts. Next, different postsynthesis treatments and their influence on the final Pt-based electrocatalysts are reviewed. Finally, perspectives on the future research and development of Pt-based electrocatalysts are provided.

## Functionalization of Carbon Supports

To improve the activity, durability, and Pt utilization efficiency, Pt-based electrocatalysts for PEMFCs are usually loaded on noncarbon or carbon supports. Noncarbon supports include nitrides, carbides, borides, mesoporous silicas, conducting polymers, and metal oxides [71, 72]. Noncarbon-supported Pt-based electrocatalysts exhibit high stability under accelerated stress testing conditions [71]. However, the low surface area and low electrical conductivity of noncarbon materials cause them to be rarely used as supports of Pt-based electrocatalysts in practical applications [71]. In comparison with noncarbon materials, carbon materials have a high surface area, suitable porosity, excellent electrical conductivity, chemical stability, and low cost, allowing a high dispersion of Pt-based nanoparticles; thus carbon materials are considered the best available supports for Pt-based electrocatalysts in practical applications [48]. Although various novel carbon materials have been reported in the literature (e.g., hollow mesoporous carbon spheres and carbon foam [19, 73–75]), most of them are still prepared at the laboratory level and impractical for mass production. Therefore, we limit our discussion to commonly used commercial carbon materials, including carbon black, graphene, carbon nanotubes (CNTs), multiwalled carbon nanotubes (MWCNTs), and carbon nanofibers. Commercial carbon black materials include Vulcan XC-72R (VXC-72R), acetylene black, Ketjen Black EC300J (EC-300), Ketjen Black EC600JD (EC-600), and Black Pearls 2000 (BP-2000). VXC-72R and acetylene black have moderate surface areas due to a small quantity of internal pores, i.e., solid carbon black. EC-300, EC-600, and BP-2000 have very high surface areas due to abundant internal pores, i.e., porous carbon black [6, 76].

As has been widely explored and accepted in heterogeneous catalysis, carbon supports not only act as simple physical supports and electron transport pathways but also have a crucial influence on the performance and durability of Pt-based electrocatalysts [77–80]. The structural properties include the surface area, pore size, size distribution, pore shape, and pore volume. The surface properties include the hydrophobicity, type and number of functional groups, and number of defects. Several previous reviews have summarized how the structure and surface properties of carbon affect nucleation and growth and thus the performance and durability of Pt-based electrocatalysts [6, 60, 81, 82]. Therefore, in this review, only a brief description, as shown below, is considered sufficient for the understanding and appreciation of the effects of the structure and surface properties of carbon on Pt-based electrocatalysts.

**Fig. 2:**
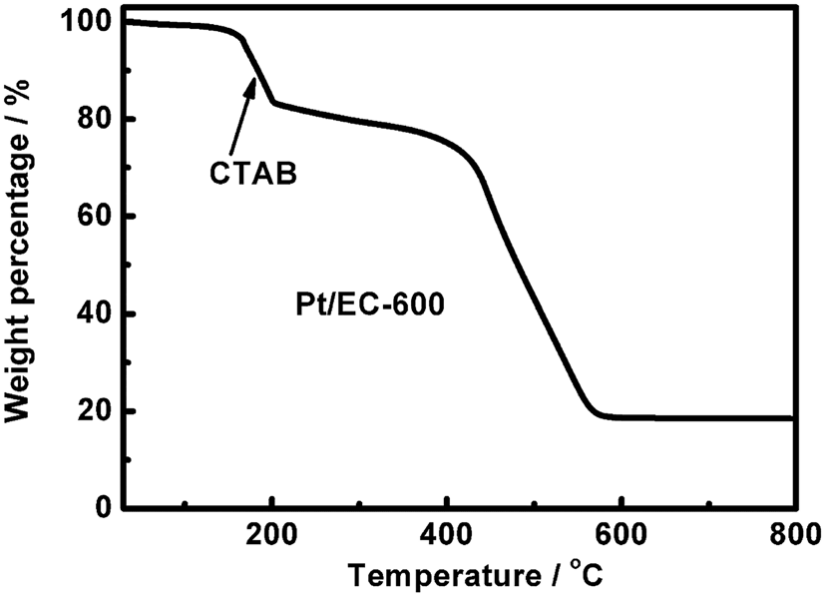
TGA curve of ~ 20 wt% Pt/EC-600 prepared by the transfer phase method (CTAB: cetyltrimethylammonium bromide)

1. *The effects of the size, size distribution, shape, and dispersion of Pt-based electrocatalysts, which influence the ECSA and activity* [76, 83–85]. For example, the surface properties of carbon can affect the interaction of carbon with solvents [86]. For instance, water, which is a polar solvent, has a very low affinity with hydrophobic carbon. Water and the Pt ions in the water can only contact the external surface of the carbon and cannot penetrate into the pores; hence, Pt-based nanoparticles are only located on the external surface of the carbon. However, acetone, which is a nonpolar solvent, can penetrate the pores of hydrophobic carbon. Therefore, Pt-based nanoparticles can be distributed on the external surface and in the pores. The affinity of carbon with polar solvents, i.e., hydrophilicity, commonly increases with an increase in the number of functional groups (such as oxygen-containing groups and nitrogen/sulfur-containing groups) and thus can be enhanced by surface functionalization [86]. In addition, surface properties can affect the interaction of carbon with Pt ions and thus affect the size and dispersion of prepared Pt-based electrocatalysts [86–88]. If the charge of Pt ions is opposite to the groups on the carbon surface, e.g., negatively charged Pt complexes (H_2_PtCl_4_, K_2_PtCl_4_, H_2_PtCl_6_) and carbon with –NH_*x*_ groups, as well as positively charged Pt complexes (Pt(acac)_2_, PtCl_2_, (NH_4_)_2_PtCl_6_, (NH_4_)_2_PtCl_4_) and carbon with –COOH or –SO_3_H groups, the Pt nanoparticles will have a small size and uniformly disperse on the carbon [86]. Otherwise, large Pt nanoparticles will be formed. For a given Pt loading, the Pt particle size generally decreases with an increase in the carbon support surface area in the one-pot synthesis method; hence, for high Pt loadings, Pt nanoparticles can still evenly disperse on carbon that has a high surface area without easily observable agglomeration compared with carbon that has a low surface area [89].
2. *The effect of the activity of Pt-based electrocatalysts*. When Pt-based nanoparticles are loaded on solid carbon, they are primarily located on the external surface of carbon, which generally leads to relatively low activity due to the poisoning of sulfonate groups in the ionomer but good local mass transport properties in the catalyst layer [76]. However, if Pt-based nanoparticles are loaded on porous carbon, most nanoparticles deposit inside the pores and show good activity, as the ionomer cannot penetrate into the small pores and contact the Pt-based nanoparticles; however, deep and complex pores will result in poor local mass transport [76]. Thus, the surface properties of carbon affect the activity of Pt-based electrocatalysts due to the metal–support interactions, including geometric and electronic effects [78, 90].
3. *The effect of the durability of Pt-based electrocatalysts* [34, 72]. The strong metal–support interaction or the formation of chemical bonds between a metal and support can improve the durability of Pt-based electrocatalysts by limiting the migration of Pt-based nanoparticles and suppressing the agglomeration of Pt-based nanoparticles [72, 91]. In addition, more stable carbon, such as highly graphitic carbon, also improves the durability of Pt-based electrocatalysts, resulting from reducing the detachment of Pt-based nanoparticles caused by carbon corrosion.
4. *The effects of the ionomer distribution on Pt/C in the catalyst layer*. The sulfonate groups of the ionomer are negatively charged. If the groups on carbon are also negatively charged, the carbon and ionomer will repel each other; otherwise, they attract each other. Such electrostatic interactions affect the size of aggregates and the ionomer distribution on Pt/C in the catalyst layer, which can further affect local mass transport. For example, if using carbon containing –NH_*x*_ as the support, the ionomer can distribute more uniformly on Pt/C, improving the mass transport at high current densities [92, 93]. Therefore, although a good dispersion of Pt-based nanoparticles can be realized on carbon with either negatively or positively charged groups by choosing appropriately charged Pt complexes as precursors, as mentioned above, the introduction of positively charged groups onto the carbon surface should be more beneficial to improve the distribution of ionomers on Pt/C than negatively charged groups (several oxygen-containing groups or –SO_3_H).
5. *The effects of the postsynthesis treatment on carbon-supported Pt-based electrocatalysts*. In our previous experiments, it was found that the carbon surface area can impact the adsorbed amount of surfactant molecules and thus influence postsynthesis treatment. For example, when using VXC-72R with a specific surface area of ~ 250 m^2^ g^−1^ as a support to prepare approximately 20 wt% (wt% means the weight percentage) Pt/C by the phase-transfer method, the surfactant molecules (such as cetyltrimethylammonium bromide (CTAB)) adsorbed on Pt/C can be removed by washing with a copious amount of hot water, and there is no weight loss peak of CTAB in the thermogravimetric analysis (TGA) curve of the resultant catalysts [30]. Similarly, using EC-600 with a specific surface area of ~ 1 270 m^2^ g^−1^ as a support of approximately 40 wt% or 60 wt% Pt/C, the adsorbed CTAB can also be removed, and no characteristic peak of CTAB in the TGA curve appears [30]. However, when using EC-600 as the support of 20 wt% Pt/C, the adsorbed CTAB molecules are difficult to completely remove even though much more hot water is used for washing as the postsynthesis treatment; thus, there is a clearly identifiable CTAB peak in the TGA curve (Fig. 2). This result is probably because compared with VXC-72R, EC-600 with a high surface area has more active sites (i.e., nucleation sites), which strongly adsorb not only Pt nanoparticles but also CTAB. If the Pt loading on carbon with a high surface area is low, only a small number of active sites are occupied by Pt nanoparticles because most active sites strongly adsorb CTAB. Otherwise, with increased Pt loading on carbon, most or even all active sites are occupied by Pt nanoparticles; thus, there is little to no strongly adsorbed CTAB, thereby simplifying postsynthesis treatment.

Regarding commercial carbon materials, the structure cannot be easily modified. However, the surface properties of carbon can be modulated by functionalization before loading Pt-based electrocatalysts to increase the number of nucleation sites or anchoring sites for the Pt-based electrocatalysts and strengthen the metal–support interactions. Functionalization methods commonly include oxidation treatment to improve the number of oxygen-containing groups, heteroatom doping, the introduction of functional groups, and the introduction of metal oxides.

### Improving Oxygen-Containing Groups

**Fig. 3:**
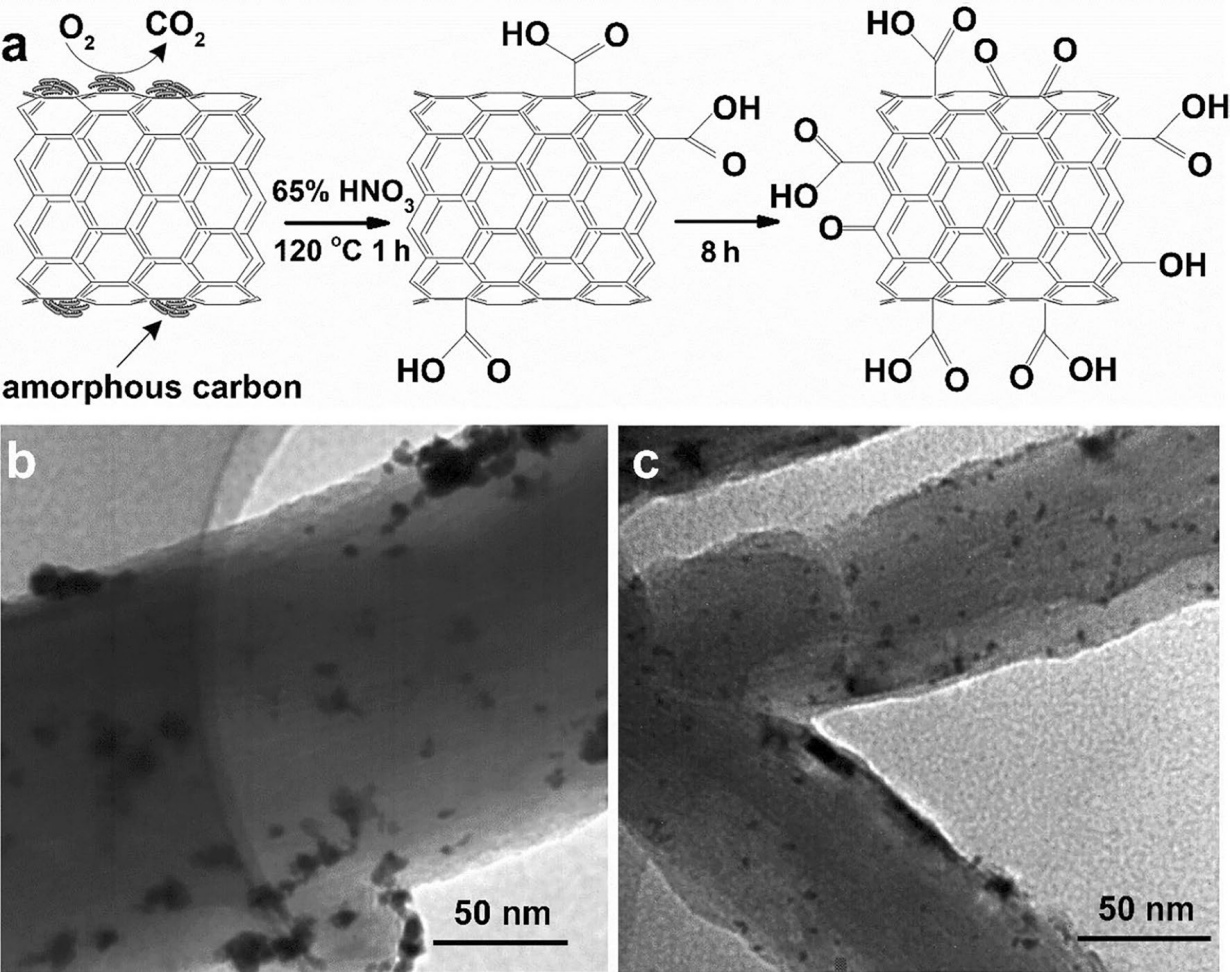
**a** Schematic of the oxidation process of MWCNTs treated with HNO_3_. Modified from Ref. [97] with permission. Copyright © 2008, Elsevier. TEM images of **b** PtRuCu/CNT and **c** PtRuCu/CNTA (CNTA: CNTs treated in 3.0 M HNO_3_ aq. at 60 °C for 3 h). Reprinted with the permission from Ref. [78]. Copyright © 2017, Elsevier

Pristine commercial carbon materials, especially carbons with a high degree of graphitization (for example, graphene or CNTs), only contain a few binding sites for anchoring metal precursors or Pt-based electrocatalysts [94, 95]. To increase the number of anchoring sites, pristine commercial carbon materials are usually functionalized by various chemical methods, among which the introduction of oxygen-containing groups is most frequently used because it is a simple but effective method. Carbon is dispersed in oxidizing acid solution (e.g., HNO_3_ and H_2_SO_4_) and then refluxed at a certain temperature for a certain period, as shown in Table 1. In addition, the carbon can be treated in an air atmosphere at an elevated temperature or ball-milled in an air atmosphere for a certain period (Table 1). During the treatment process, the C–C bonds on the carbon surface are attacked by the oxidants and then broken, forming dangling bonds or oxygen-containing groups by bonding carbon with oxygen atoms (Fig. 3a). Based on infrared radiation (IR) spectroscopy or X-ray photoelectron spectroscopy (XPS), oxygen-containing groups can be formed on the carbon surface, such as carboxylic, hydroxyl, lactone, phenol, epoxy, carbonyl, anhydride, ether, or quinone groups. The dangling bonds and oxygen-containing groups act as nucleation sites or anchoring sites for Pt-based electrocatalysts via electrostatic, coordinative, or van der Waals interactions [60, 78, 96–98]. Therefore, Pt-based electrocatalysts loaded on oxidation-treated carbon present better dispersion and fewer agglomerates than those loaded on pristine carbon (Fig. 3b and c). In addition, the oxygen-containing groups increase the hydrophilicity of the carbon, i.e., the affinity of carbon with polar solvents, thereby improving its dispersion in polar solvents, which can promote the even adsorption of metal ions on the carbon surface. It also improves the dispersion of carbon-supported Pt-based electrocatalysts in ink (commonly using a mixed solvent of water and ethanol/isopropanol, which are polar solvents) and the quality of the fabricated catalyst layer [99].

**Table 1:** Oxidation treatment methods of carbon materials (arranged by the type of carbon)

Methods	Carbon	Conditions	Ref.
Liquid activation	VXC-72R	5% HNO_3_, 0.07 M (1 M = 1 mol L^−1^) H_3_PO_4_, and 0.2 M KOH aq., refluxed at 120 °C for 16 h	[272]
	EC-300, VXC-72R, or BP-2000	6 M HCl to dematerialize metal impurities, and then oxidated in 70% HNO_3_ for 7 h	[273]
	VXC-72R, EC-600, BP-2000, EC-300, or MWCNTs	3.0 M or concentrated HNO_3_ solution, refluxed at 60, 70, 80, or 120 °C for 1–8 h	[15, 78, 97, 274, 117]
	MWCNTs, or VXC-72R	Concentrated H_2_SO_4_ and HNO_3_, refluxed at 60 °C for 1 h or stirred at room temperature for 30 min	[98, 275]
	CNTs	4.0 M H_2_SO_4_, refluxed at 80 °C for 4 h	[276]
Heat treatment	MWCNTs	Calcining at 425–550 °C in air for 1–120 min	[97]
Ball milling	MWCNTs	Ball milling in air for 60 h	[97]

However, the presence of dangling bonds and oxygen-containing groups largely destroys the graphitic structure on a carbon surface. This structural destruction decreases the conductivity and accelerates the corrosion of carbon supports, causing low output performance and durability for carbon-supported Pt-based electrocatalysts in PEMFCs. Due to the decrease in area of the graphite regions, the conductivity of VXC-72R carbon black and MWCNTs can be reduced by a factor of three after pretreatment, resulting from the partial damage of the conjugated sp^2^ network [78]. After being bonded with oxygen during the oxidation treatment, the carbon atom hybridization changes from sp^2^ to sp^3^. The increased number of oxygen-containing groups and the decreased degree of graphitization will accelerate the electrochemical oxidation of carbon, especially when fuel starvation or PEMFC startup/shutdown events occur, which is of prime importance for the long-term stability of Pt-based electrocatalysts [100, 101]. To remove the formed oxygen-containing groups on MWCNTs after loading Pt nanoparticles, the MWCNTs are again heat-treated at 700 °C. However, during the thermal treatment, the Pt nanoparticles increase in size due to Ostwald ripening or coalescence [97].

To overcome the above issues, several recent studies [88, 102, 103] have focused on developing other methods to functionalize carbon materials, including heteroatom doping, the introduction of functional groups, or the introduction of metal oxides. These methods not only increase the number of binding sites for Pt-based electrocatalysts but also boost the activity and durability of electrocatalysts due to enhanced metal–support interactions.

### Heteroatom Doping

**Fig. 4:**
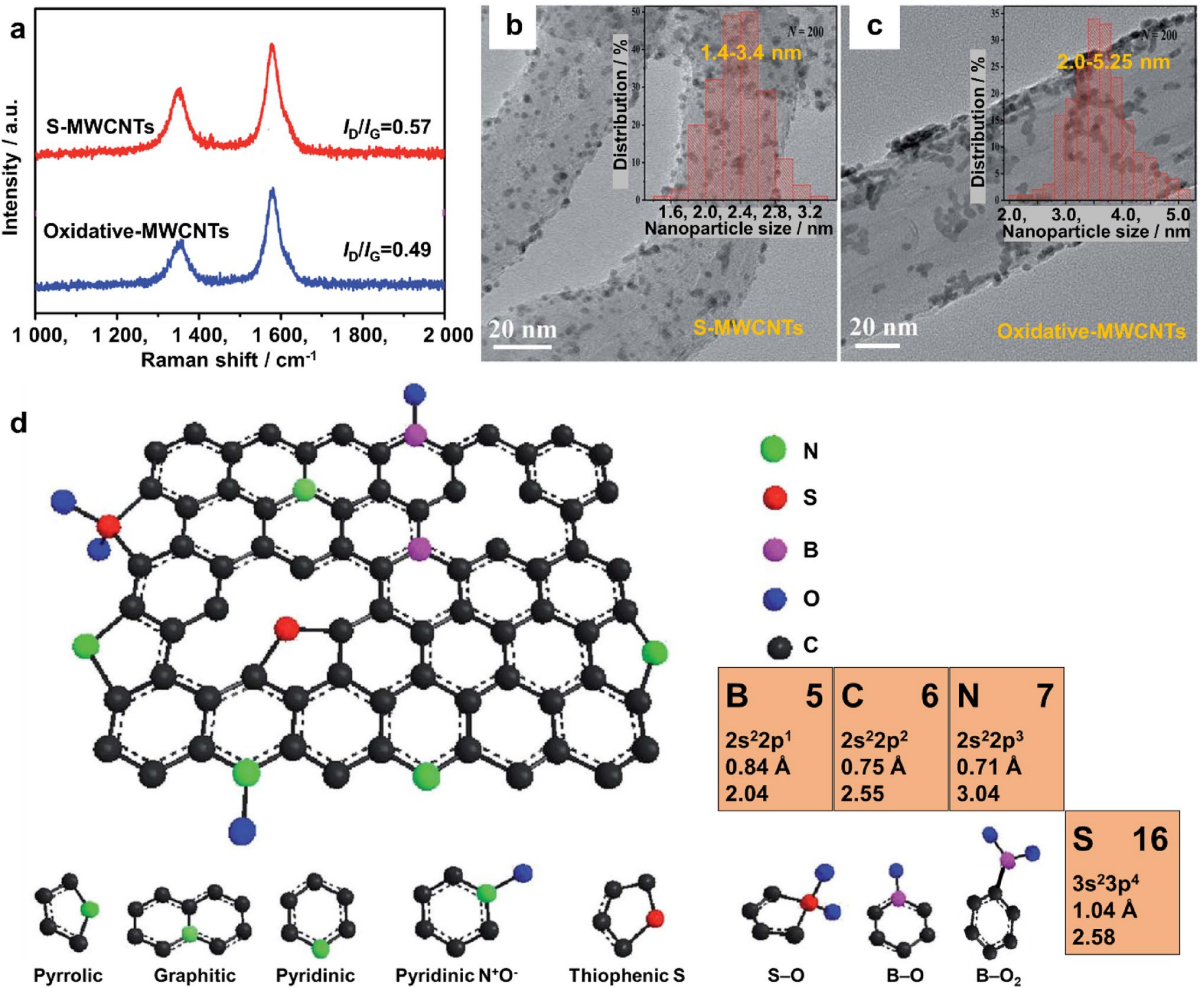
**a** Raman spectra of sulfur-doped MWCNTs (S-MWCNTs) and oxidized-MWCNTs. TEM images of **b** Pt supported on S-MWCNTs or **c** oxidized-MWCNTs, the insets: particle size distribution histogram. Adapted with permission from Ref. [107]. Copyright © 2017, Royal Society of Chemistry. **d** Schematic of heteroatom-doped reduced graphene oxide (RGO). 1 Å = 1 × 10^−10^ m. Adapted with permission from Ref. [105]. Copyright © 2016, Royal Society of Chemistry. The atomic data of B, C, N, and S, including the atomic number, outer electron configuration, covalent radius, and electronegativity are referred to Ref. [213]

The surface physicochemical properties of carbon are modulated by doping heteroatoms that have different electron configurations, atomic sizes, and electronegativities than carbon; commonly used dopants include N, S, and B [104–106]. Due to the different electron configurations and atomic sizes of carbon, the incorporation of N, S, or B into a carbon matrix induces more structural defects and more edge plane exposure, as demonstrated by the increased *I*_D_/*I*_G_ ratio (*I*_D_ is the intensity of the peak assigned to defects, *I*_G_ is the intensity of the peak assigned to the graphitic structure) that is obtained by Raman spectroscopy; therefore, more nucleation sites or anchoring sites for Pt-based electrocatalysts are produced [107, 108] (Fig. 4a–c). Because of its different electron configurations and electronegativities, the electronic properties of carbon are modulated by doping N, S, or B, which further influences the electronic properties of supported Pt-based electrocatalysts. According to the XPS data, electrons transfer from Pt-based electrocatalysts to N-doped carbon due to the higher electronegativity of N [102]. Electrons transfer from B-doped carbon to Pt-based electrocatalysts due to the lower electronegativity of B [109]. This can change the adsorption strength of the reaction intermediates on Pt-based electrocatalysts, thus boosting electrocatalytic activity [108, 110]. The strong metal–support interaction also enhances the durability of Pt-based electrocatalysts with heteroatom-doped carbon as a support in PEMFCs [111]. In short, the doping of heteroatoms contributes to a more homogeneous dispersion of Pt-based electrocatalysts on carbon and improves the activity and durability of Pt-based electrocatalysts.

The methods of doping heteroatoms into carbon can be divided into two categories: in situ and ex situ doping methods [112, 113]. Regarding in situ doping methods, heteroatom doping is conducted during the synthesis of carbon materials, for example, using heteroatom-containing materials as the carbon precursor for chemical vapor deposition or directly carbonizing heteroatom-containing materials [19, 114–116]. In situ doping methods will not be considered further in this review because the emphasis is the functionalization of commercial carbon. Regarding ex situ doping methods, heteroatom doping is generally performed by annealing a mixture of heteroatom-containing materials and commercial carbon after oxidation treatment or annealing oxidized carbon in an NH_3_/H_2_S atmosphere. Several ex situ doping methods for preparing N-, S-, and B-doped carbon and the amounts of doped heteroatoms are summarized in Table 2. The amount of doped heteroatoms in carbon can be controlled to some extent by regulating the reaction conditions, such as the annealing temperature and the concentration and nature of the precursor [102, 117–120]. For example, the amount of doped heteroatoms first increases and then decreases with increase in annealing temperature [117, 118]. From XPS studies, the N species in the carbon matrix include pyrrolic N, pyridinic N, graphitic N, or pyridine-N-oxide [120], while the S species in the carbon matrix include thiophene or –SO_*x*_ [121], and the B species in the carbon matrix include BC_3_ (B-doped carbon), BC_2_O, or BCO_2_ [122] (Fig. 4d).

**Table 2:** Summary of ex situ doping methods for preparing N-, S-, and B-doped carbon and the amount of doped heterotatoms (arranged by the type of carbon)

	Carbon	Doping methods	Amount of doped heteroatoms	Refs.
N	Carbon black	Annealing a mixture of preoxidized carbon (1.0 g), 1,10-phenanthroline (0.5 g), and NiCl_2_·6H_2_O (0.24 g) at 800 °C in N_2_ for 2 h	–	[277]
	VXC-72R	Mixing carbon with urea (the weight ratio is 1/1.5) by grinding, then heating at 300 °C for 2 h in air	9.8 at% (at% means the atomic percentage)	[278]
	EC-300	Annealing preoxidized carbon at 200, 400 or 600 °C in NH_3_ and Ar for 2.5 h	0.84, 1.04, 0.81 at% (200–600 °C)	[117]
	EC-600	Mixing oxidized carbon and melamine (the weight ratio is 1/1), then treating at 800 °C in Ar for 2 h	2.1 at%	[279]
	Graphene	Annealing a mixture of GO and urea (the weight ratios are 1/1, 1/2, 1/3), at 700 °C in N_2_ for 2 h	8.19, 8.5, 9.9 at% (1/1, 1/2, 1/3)	[102]
	Graphene	Annealing a mixture of GO and dopamine at 800, 1 000, or 1 200 °C in Ar for 2 h	3.79, 3.2, 2.78 at% (800–1 200 °C)	[280]
	Graphene	Annealing a mixture of GO and dicyandiamide (the weight ratio is 1/20) at 800 °C in N_2_ for 2 h	6–7 at%	[281]
	Graphene	In the presence of EDACM, grafting various amines ^a^ onto GO, then treating at 800 °C in Ar for 1 h	0.72–4.3 at% for various amines	[120]
S	CNTs	Mixing CNTs and thiourea (the weight ratios are 2/1, 1/1, 1/2), then treating at 800 °C in Ar for 3 h	0.69, 0.83, 0.98 at% (2/1, 1/1, 1/2)	[119]
	MWCNTs	In the presence of SDS and (NH_4_)_2_S_2_O_8_, EDOT polymerizes on oxidized MWCNTs, and then, the PEDOT-MWCNTs are treated at 800 °C in N_2_ for 3 h	0.31 at%	[107]
	MWCNTs, graphene	Annealing a mixture of GO or oxidized MWCNTs and phenyl disulfide (the weight ratio is 2/1), at 1 000 °C in Ar for 30 min	2.32, 1.02 at%	[121] [98]
	Graphene	Annealing graphene at 600, 700, 800, 850, 900 °C in carbon disulfide (CS_2_) vapor with Ar gas for 1 h	0.73, 0.94, 1.79, 2, 1.04 at% (600–900 °C)	[118]
	Graphene	Heating RGO at 800 °C in H_2_S for 3 h (1 000 ppm (1 ppm = 1 mL m^–3^) of H_2_S balanced in N_2_, 150 mL min^–1^)	3 at%	[282]
	Graphene	Annealing a mixture of GO and benzyl disulfide in Ar at 600–1 050 °C	1.53, 1.35, 1.3 wt% (600, 900, 1 050 °C)	[283]
	Graphite	Ball milling pristine graphite and sulfur (S8) (the weight ratio is 1/4) at 500 r min^−1^ for 48 h	4.94 at%	[284]
B	Graphene	Annealing a mixture of GO and H_3_BO_3_ (the weight ratio is 1/4) at 900–1 000 °C in H_2_/Ar (5%/95%) for 2–4 h, then removing residual H_3_BO_3_	3.2 at% (900 °C, 4 h)	[108, 122, 285]

^a^Various amines (ethylene diamine, diethylene triamine, triethylene tetramine, tetraethylene pentamine, or pentaethylene hexamine)

GO: graphene oxide; EDACM: *N*-ethyl-*N*-(3-dimethyl aminopropyl)carbodiimide methiodide; EDOT: 3,4-ethylenedioxythiophene; SDS: sodium dodecyl sulfate

### Introduction of Functional Groups

Carbon can also be functionalized by the introduction of thiol (–SH), amine (–NH_*x*_), or carboxyl (–COOH) groups, as well as groups, molecules, or polymers coupled with –SH, –NH_*x*_, or –COOH terminal moieties. To introduce functional groups onto a carbon surface, two methods have been developed, namely covalent grafting and noncovalent interaction.

**Fig. 5:**
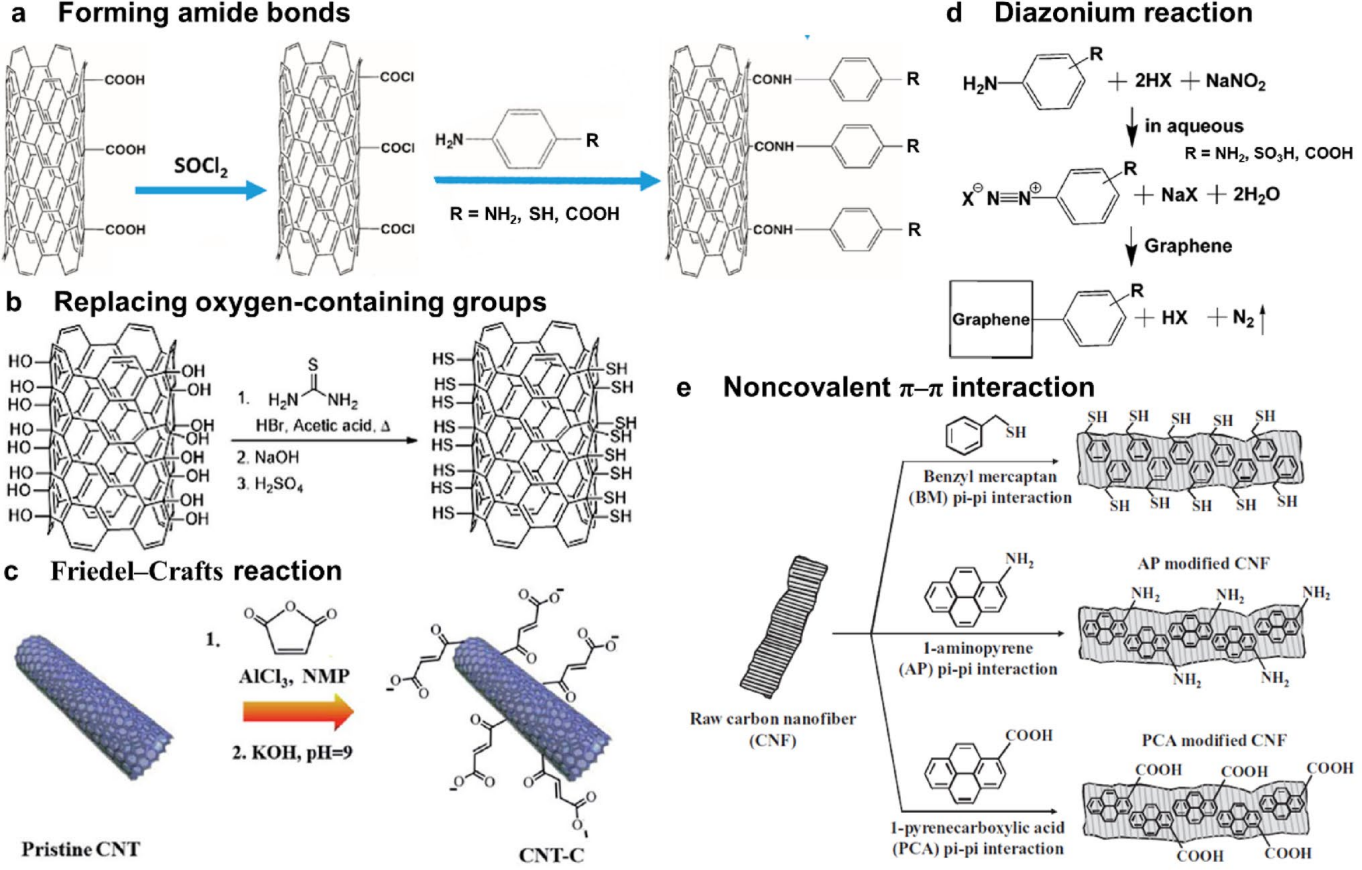
Procedures for functionalizing carbon materials by covalent grafting, e.g., **a** forming amide bonds (reprinted with permission from Ref. [126], copyright © 2016, American Chemical Society), **b** replacing oxygen-containing groups with –SH (adapted with permission from Ref. [123], copyright © 2014, Royal Society of Chemistry), **c** the Friedel–Crafts reaction (reprinted with permission from Ref. [84], copyright © 2015, American Chemical Society), **d** the diazonium reaction (reprinted with permission from Ref. [125], copyright © 2016, American Chemical Society), and **e** the noncovalent interaction, i.e., π–π interaction (adapted with permission from Ref. [128], copyright © 2011, Wiley–VCH)

Covalent grafting, i.e., forming covalent linkages between carbon and functional groups, can be realized by replacing oxygen-containing groups with functional groups such as –SH or –NH_2_ [123], forming amide bonds (–CONH–) between oxidized carbon and amine-terminated functional molecules [124], the diazonium reaction [125] or the direct Friedel–Crafts reaction [84] (Fig. 5a–d and Table 3). In these methods, carbon commonly needs to be oxidized to yield abundant oxygen-containing groups on the surface before grafting functional groups. The graphite structure on the carbon surface is broken during the oxidation treatment, as mentioned above; therefore, the process of grafting functional groups on the carbon support decreases its conductivity and long-term stability [126]. Notably, carbon needs to undergo oxidation treatment to effectively achieve heteroatom doping; however, heteroatom doping has less impact on the conductivity and durability of carbon than the grafting of functional groups. Annealing at an elevated temperature is commonly needed for heteroatom doping, which may help to recover the graphitic structure of carbon to some extent; furthermore, only a small number of heteroatoms are introduced into the carbon matrix, causing less impact on the structural integrity of graphitic carbon.

**Table 3:** Summary of the methods for introducing functional groups onto carbon (arranged by the type of carbon)

Methods	Carbon	Chemical materials and conditions	Amount	Refs.
Covalent grafting	VXC-72R-NH_*x*_	Annealing oxidized carbon at 200 °C for 4 h in pure NH_3_ gas (1 L min^–1^)	0.9 wt% (N)	[93]
	MWCNT-COOH/NH_2_/SH	MWCNT-Cl and ATP, PPDA, or ABA in toluene, stirred at 70 °C for 24 h	–	[126]
	MWCNT-COOH	MWCNTs in toluene containing AIBN, refluxed at 75 °C for 4 h, and then placed in a mixture of NaOH and methanol and refluxed at 70 °C for 48 h	5.1 wt%	[286]
	CNT-SH	CNT-OH and thiourea in HBr and acetic acid, stirred for 48 h, and then placed in NaOH overnight	6.55 at% (S)	[123]
	CNT-COOH	CNT, MAH, and AlCl_3_ in dried NMP, refluxed at 90 °C in N_2_ for 4 h, and then at 150 °C for 48 h	–	[84]
	Graphene-*p*-phenyl-NH_2_/SO_3_H	Dispersing RGO in deionized water, then adding sulfanilic acid, concentrated H_2_SO_4_ (or PPDA, concentrated HNO_3_), and NaNO_2_ before keeping the solution at 60 °C for 4 h	5.7 at% (N), 1.3 at% (S)	[125]
Noncovalent interaction	VXC-72R/MWCNT-polydopamine	Dissolving carbon in tris-HCl buffer solution and then adding dopamine hydrochloride, stirred at room temperature for 24 h	–	[287]
	MWCNT-PDDA	MWCNTs (200 mg) and PPDA (40 mg) in isopropanol, stirred for 12 h	–	[88]
	MWCNT-polyaniline-SH	MWCNTs-polyaniline in 2,5-dimercapto-1,3,4-thiadiazole and H_2_SO_4_/ethanol, stirred in the microwave reactor for 5 min	–	[288]
	COOH, NH_*x*_ or SH– terminated MWCNTs	MWCNTs and ABA, ATP or PPDA in a mixture of ethanol/toluene or dehydrated toluene and sonicated for 3–4 h	–	[126]
	CNT-PDDA/PEI/AP/THF	CNTs in PDDA/PEI aq., AP ethanol solution or THF, stirred overnight	3.5, 3, 1 wt% (PDDA, PEI, AP/THF)	[87]
	CNT-polyaniline	Oxidized CNTs and the aniline monomer in acidic solution, then adding (NH_4_)_2_S_2_O_8_ and polymerizing at 0–5 °C for 5 h	–	[289]
	Graphene-nitrobenzene	Graphene in NBDTFB aq. and stirred for 30 min	–	[290]
	Carbon nanofibers	Raw CNFs and BM, AP, or PCA in ethanol and refluxed at 25 °C for 3 h	10 wt%	[128]

ATP: 4-aminothiophenol; PPDA:* p*-phenylenediamine; ABA: 4-aminobenzoic acid; AIBN: azodiisobutyronitrile; MAH: maleic anhydride; NMP: *N*-methyl-2-pyrrolidone; BM: benzyl mercaptan; AP: 1-aminopyrene; PCA: 1-pyrenecarboxylic acid; PDDA: poly(diallyldimethylammonium chloride); PEI: polyethylenimine; THF: tetrahydrofuran; NBDTFB: 4-nitrobenzenediazonium tetrafluoroborate

Molecules or polymers with functional groups can be introduced onto the carbon surface by noncovalent interactions and π–π interactions between the pyrene, phenyl or C = C moieties of the molecules or polymers and the graphite structure of carbon materials, as shown in Fig. 5e. Briefly, π–π interactions are a type of physisorption and electrostatic interaction that does not destroy the graphitic structure of carbon materials. Raman spectroscopy shows that the *I*_D_/*I*_G_ ratio for the as-prepared *p*-phenylenediamine-functionalized MWCNTs is 0.93, similar to pristine MWCNTs (0.95), meaning that the structural integrity of MWCNTs is preserved while being functionalized [88]. Moreover, compared with covalent grafting methods, noncovalent interaction approaches are generally simpler, needing only to physically mix carbon with molecules or polymers that have the desired functional groups or in situ polymerizing monomers on the surface of carbon (Table 3). However, compared with covalent bonds, π–π stacking is an intermolecular interaction; thus, it is a weaker interaction that may be unstable during the long-term operation of PFMFCs.

The introduced groups, molecules or polymers play a role as interlinkers between the Pt-based electrocatalysts and carbon. Therefore, the functional groups should be close to the carbon surface. A long and flexible chain terminated with functional groups may give rise to a large contact resistance between Pt-based electrocatalysts and carbon, affecting the electron transport pathway and decreasing the activity of carbon-supported Pt-based electrocatalysts [84, 88, 127]. Furthermore, functional groups such as –SH or –NH_*x*_ commonly have a strong interaction with Pt-based electrocatalysts, probably poisoning them and decreasing their electrocatalytic activity [128].

### Introduction of Metal Oxides

While carbon materials have many advantages as supports for Pt-based electrocatalysts, they are easily corroded under PEMFC conditions, e.g., a high oxygen concentration and a cell voltage of approximately 1.0 V under no-load operation or approximately 1.4–1.6 V during start–stop operation or fuel starvation [129, 130]. Carbon corrosion will cause the detachment of Pt-based electrocatalysts and increase the hydrophilicity of the catalyst layer, resulting in flooding and the production of CO, which can poison Pt-based electrocatalysts [131]. Moreover, the affinity of Pt-based electrocatalysts with carbon is weak, easily resulting in the migration and coalescence of Pt-based electrocatalysts [132]. Compared with carbon materials, metal oxides, such as indium tin oxide (ITO) [133], CeO_2_ [134], TiO_2_ [135], SnO_2_ [136], NbO_*x*_ [137], WO_3_ [138], and Mn_3_O_4_ [139], commonly have higher stabilities in oxidative and high-potential environments. The strong metal–metal oxide interaction can effectively mitigate the degradation of Pt-based electrocatalysts. However, metal oxides cannot meet the support requirements of Pt-based electrocatalysts due to their low surface area, electronic conductivity, and performance [137]. Alternatively, neither carbon materials nor metal oxides satisfy both the activity and durability of carbon-supported Pt-based electrocatalysts for PEMFCs. Thus, carbon–metal oxide composite supports may be promising and are expected to be able to exhibit the properties of both metal oxides and carbon materials, i.e., a high surface area, sufficient electrical conductivity, and high stability in PEMFCs, and the strong metal–support interaction [132].

Table 4 shows some methods for preparing carbon–metal oxide composite supports by depositing metal oxides as particles onto carbon. Pt-based electrocatalysts with carbon–metal oxide composite supports generally show improved electrocatalytic activity [140] and enhanced durability [129, 133] due to the enhanced metal–support interaction. Metal oxide nanoparticles also act as a physical barrier, which decreases the chance of Pt-based nanoparticles encountering each other to a certain extent, thereby enhancing the durability. In addition, the CO tolerance of Pt-based electrocatalysts is improved because the CO adsorbed on Pt-based electrocatalysts can be easily oxidized by the oxygen-containing species that are supplied by the metal oxides [133, 134, 140].

**Table 4:** Summary of the methods for introducing metal oxides onto carbon (arranged by the type of carbon)

Composite supports	Methods	Loading, size	Refs.
ITO/VXC-72R	Cathodic arc deposition	12.2 wt%, 5.6 nm	[133]
SnO_2_/VXC-72R	Electrochemical deposition	4 wt% (Sn), 2.9 nm	[141]
WO_2_/VXC-72R	Mixing pre-prepared WO_3_ with carbon, followed by evaporating the solvent under vacuum at 80 °C	12.5 wt%, –	[138]
CeO_2_/C	Ce(NO_3_)_3_ and carbon are mixed in water, autoclaved, and heated at 160 °C for 8 h	3 wt%, –	[134]
NbO_*x*_/acetylene black	Sol–gel method by using Nb(OCH_2_CH_3_)_5_ as the precursor	5 wt% or 12 wt%, –	[137]
CeO_2_/MWCNTs	MWCNTs, Ce(NO_3_)_3_ and urea in ethylene glycol and then heated at 130 °C for 3 h	0.8 wt%, 3–5 nm	[129]
TiO_2_/MWCNTs	Atomic layer deposition	–	[135, 291]
Mn_3_O_4_/CNTs	Hydrolysis of (CH_3_COO)_3_Mn on the oxidized CNTs	–	[139]
ITO/graphene	RGO, In(acac)_3_ and tin tert-butoxide are mixed in benzyl alcohol, autoclaved, and heated at 200 °C for 17–24 h	75 wt%, –	[130, 132]
SnO_2_/graphene	GO, urea, and SnCl_2_ are mixed in water and then irradiated in a microwave furnace at 180 °C for 15 min	11, 22.3, 38 wt%; 3.18, 2.97, 3.24 nm	[136]
N-doped graphene-TiO_2_	N-doped TiO_2_ and N-doped graphene are mixed in water, autoclaved, and then heated at 120 °C for 3 h	–	[103]
TiO_2_/graphene	Graphene, SDS, TiCl_3_, and H_2_O_2_ are mixed in water, stirred at 90 °C for 16 h, and then washed, dried, and treated in H_2_ at 400 °C for 2 h	–	[292]

SDS: sodium dodecyl sulfonate

**Fig. 6:**
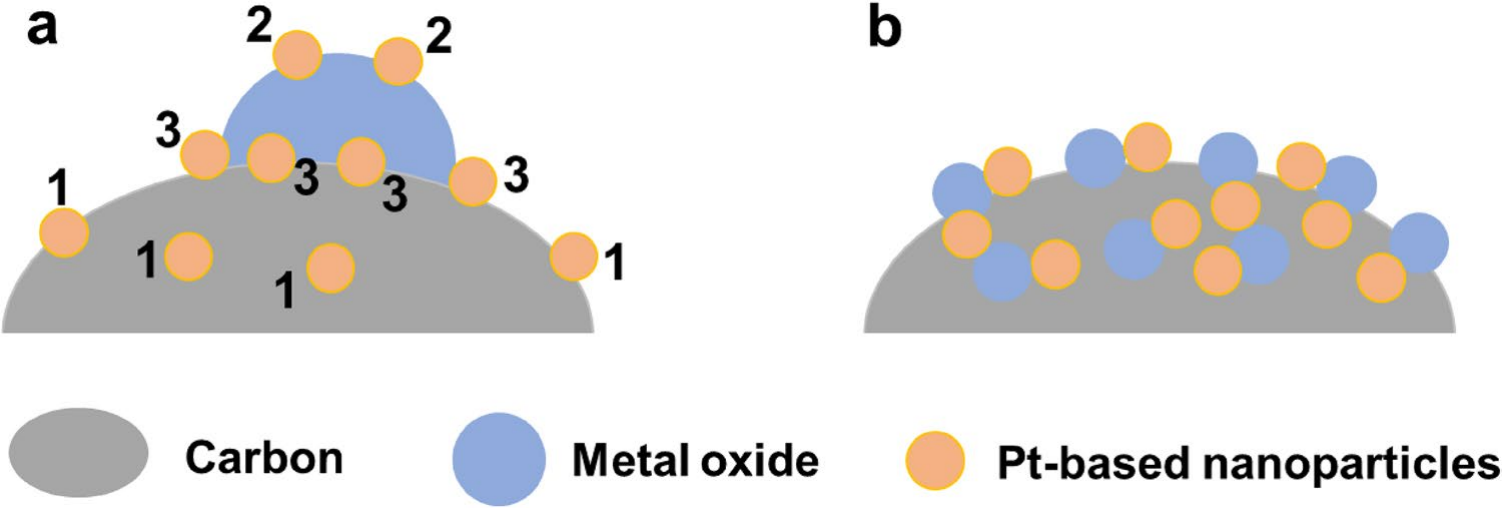
Schematic showing the location of Pt-based nanoparticles deposited on carbon–metal oxide composite supports when metal oxide particles are **a** large and **b** small

Figure 6a exhibits three scenarios for Pt-based nanoparticles deposited on composite supports with a large metal oxide particle size, i.e., only on the carbon surface (particle 1), only on the metal oxide surface (particle 2) or at junctions between metal oxide particles and carbon (Pt-based nanoparticles that simultaneously contact carbon and metal oxides to form triple junctions, particle 3). Metal oxides may have no influence on the performance of particle 1, and particle 2 likely has no electron channel. The potential advantages of carbon–metal oxide composite supports may be embodied only for particle 3. To maximize the advantages of carbon–metal oxide composite supports, the number of junctions should be increased by improving the metal oxide dispersion or decreasing the metal oxide particle size (similar to Pt nanoparticles) [141] (Fig. 6b). However, it remains a challenge to deposit small metal oxide particles on carbon.

As discussed earlier, each of the four functionalization methods for carbon materials has advantages and disadvantages, and a detailed comparison of the four functionalization methods is summarized in Table 5. The selection of suitable methods should depend on the requirements of practical cases.

**Table 5:** Comparison of the four functionalization methods of carbon materials

Methods	Advantages	Disadvantages
Oxidation treatment	Simple	Destroys the graphitic structure on the carbon surface Reduces the conductivity Reduces the stability of carbon
Heteroatom doping	Strong metal–support interaction	Low doping amount with the ex situ doping method
Introduction of functional groups	Covalent grafting: stable due to covalent bond; Noncovalent interaction: simple Complete graphitic structure	Long and flexible chain: a large contact resistance Functional groups, such as –SH or –NH_*x*_, may poison Pt Covalent grafting: destroys the graphitic structure Noncovalent interaction: π–π stacking, a weaker interaction, unstable
Introduction of metal oxides	More stable CO tolerance	Depositing small metal oxide particles is still challenging

## Methods for Loading Pt-Based Electrocatalysts on Carbon Supports

The effective integration of Pt-based electrocatalysts with carbon supports is crucial to obtain high-quality electrocatalysts, and the experimental methods commonly used can be classified into two categories based on when the carbon supports are added (i.e., before or after the formation of Pt-based nanostructures): one-pot synthesis (in situ mixing) and ex situ mixing methods [60, 85].

### One-Pot Synthesis Method

In the one-pot synthesis method, carbon is directly added into the reduction system, and carbon-supported Pt-based electrocatalysts are formed when the reaction finishes; this all occurs in only one step, which can reduce the cost associated with electrocatalysts to some degree [8]. During the reaction process, Pt-based nanostructures may directly nucleate or grow on carbon, forming chemical bonds with carbon through the functional groups or dangling bonds on the surface, thereby enhancing the stability of Pt-based electrocatalysts [142, 143]. However, the shape of Pt-based nanostructures may not be easily controlled due to the interference of carbon supports during the nucleation and growth process [85], which may be a reason why ex situ mixing methods are more commonly used than one-pot synthesis methods in the reported literature, especially for nonspherical carbon-supported Pt-based electrocatalysts. For example, if no carbon is present, Pt nanowire networks are prepared by reducing Pt complexes that are confined in a worm-like reverse micellar network. However, when the addition of carbon and other conditions are kept the same, only spherical Pt nanoparticles are formed on carbon due to the formation of spherical reverse micellar particles adsorbed on carbon [30, 144]. Moreover, the organic molecules used in the reaction solution, e.g., ligands of metal precursors, solvents, or structure-capping agents, may adsorb easily on the carbon surface, especially those with high surface areas, and these molecules are difficult to remove. This may be another reason for the lack of one-pot synthesis methods used to prepare nonspherical carbon-supported Pt-based electrocatalysts whose preparation commonly needs various structure-capping agents to control the shape or to be carried out in an organic solvent.

### Ex Situ Mixing Method

In the ex situ mixing method, Pt-based electrocatalysts are loaded onto carbon by physically mixing prepared Pt-based electrocatalysts with carbon. Regarding the ex situ mixing method, the size or shape of the Pt-based nanostructures can be easily controlled without the reaction system disturbing the carbon [68, 85]. However, during mixing, Pt-based electrocatalysts are loaded onto carbon by physisorption, likely resulting in a weak interaction between the Pt-based electrocatalysts and carbon and thus inferior stability. Moreover, the Pt-based nanostructures tend to aggregate on carbon due to hydrogen bonds between the residual surfactants on the surface of the nanostructures. Unlike the one-pot synthesis method in which the metal loading on carbon can be easily increased by increasing the ratio of Pt precursor and carbon [30], carbon-supported Pt-based electrocatalysts with high metal loading are rarely prepared by the ex situ mixing method because the amount of loaded Pt barely increases with an increasing ratio of Pt-based electrocatalysts to carbon after reaching a certain critical value [85].

Compared with the ex situ mixing method, the one-pot method may be more suitable for fabricating supported Pt-based electrocatalysts with high metal loading, high dispersion, and good stability, making it more suitable for the mass production of Pt-based electrocatalysts (Table 6).

**Table 6:** Comparison of one-pot and ex situ mixing methods

Methods	Advantages	Disadvantages
One pot	Suitable for high metal loading on the support Promoting the dispersion of metal on the support Good stability: strong metal–support interaction Suitable for mass production	Hard to control the shape
Ex situ mixing	Easy control of the size and shape	Hard to obtain a high metal loading on the support Bad dispersion of metal on the support Weak metal–support interaction Unsuitable for mass production

## Synthesis of Pt-Based Electrocatalysts

Regarding Pt-based electrocatalysts, controlling the size, size distribution, and shape is crucial to their activity and durability, as mentioned in the Introduction. Generally, the formation of Pt-based electrocatalysts involves two stages: nucleation and growth. During nucleation, building blocks, such as metal atoms, metal-ion dimers, or trimers (such as Pt(II)-Pt(I) or Pt(I)-Pt(I) dimers [145, 146]), aggregate into nuclei (small clusters) and then form seeds once the nuclei grow over a specific size. During growth, the seeds gradually grow into the final nanocrystals through the addition of atoms. The eventual size and shape of the nanocrystals are simultaneously governed by the complex processes of nucleation and growth, which experimentally depend on the types and concentrations of metal precursors, reducing agents, and structure-directing agents, as well as the solvents and reaction conditions.

**Fig. 7:**
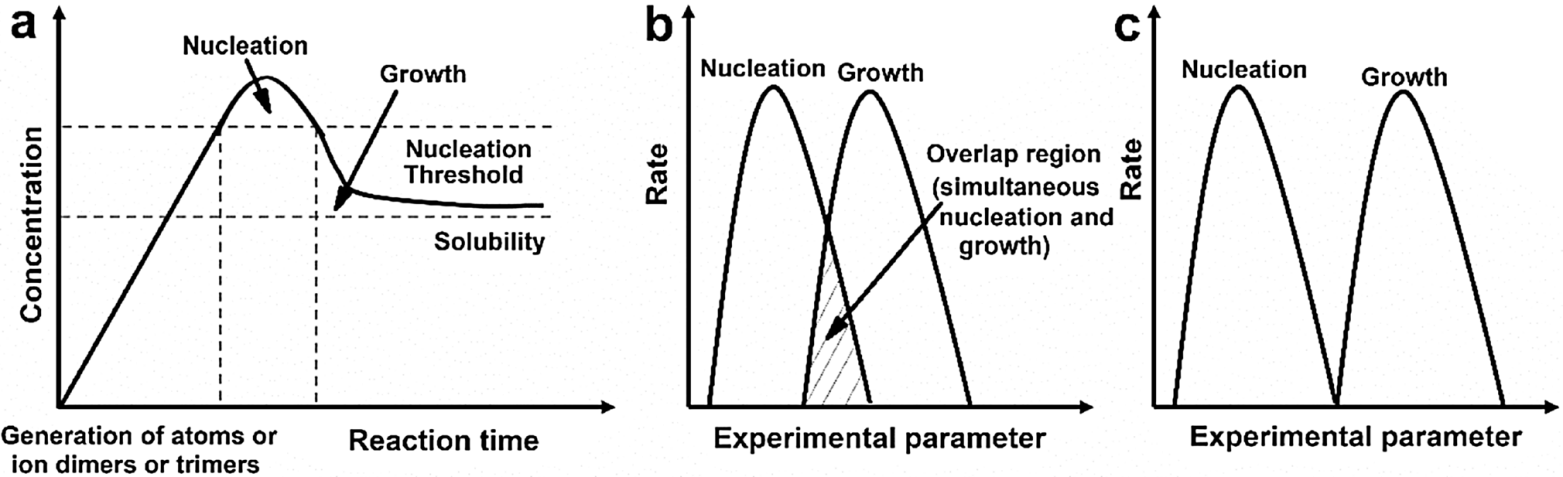
**a** Plot of the concentration of building blocks against the reaction time. Adapted with permission from Ref. [147]. Copyright © 1950, American Chemical Society. **b** The case of nucleation overlapping with growth, i.e., new seeds are formed when previously formed seeds are growing, causing different growth periods for seeds produced at different times. **c** Ideal case in which nucleation and growth are completely separated, leading to nearly the same growth period for all seeds. Adapted with permission from Ref. [271]. Copyright © 2012, Springer

During nucleation and growth, the concentration of the building blocks changes with the reaction time, which is often presented in a classical Lamer’s plot [147]. As shown in Fig. 7a, when the concentration of the building blocks becomes much larger than the nucleation threshold, abundant nuclei or seeds are formed; thus, the concentration of the building blocks drops quickly below the nucleation threshold and inhibits additional nucleation events. Therefore, a short burst of nucleation or the formation of a large number of seeds in a very short time that is then followed by gradual growth can yield small and uniform nanocrystals, owing to every seed having nearly the same growth period. In other words, nucleation and growth are nearly separated or only have a small overlap, as shown in Fig. 7b and c. In contrast, slow, continuous nucleation over the entire reaction time gives rise to a broad size distribution due to variations in the growth period of seeds produced at different times. To force nucleation to occur in a short time, there are several effective strategies in real synthesis processes, e.g., changing the type or improving the concentration of Pt precursors [60, 62], using reducing agents with strong reducing capacity [65] or increasing the concentration of reducing agents [148, 149], increasing the reaction temperature [68], changing the solvent [150], and increasing the number of nucleation sites on carbon supports for the one-pot synthesis method [151]. However, burst nucleation consumes most of the precursor, likely resulting in fewer shape-controlled Pt nanocrystals due to insufficient feedstock for growth [68].

In addition to the particle size and size distribution, the nucleation process also affects the final shape because the formed seeds may have a well-established shape, such as single crystal, singly twinned, multiply twinned, or stacking fault-lined structures. For example, a single crystal seed will grow into an octahedron, cuboctahedron or cube, a multiply twinned seed will grow into a decahedron or icosahedron [152], and a seed with stacking faults will grow into plates [153]. More details regarding the factors determining the shape of seeds can be found elsewhere, for example, in Xia’s review [154].

The shape of Pt-based electrocatalysts depends on not only the shape of the seeds but also the growth process. During growth, the deposited atom diffuses around the seed surface until it meets a low-energy site. If the diffusion rate of atoms is larger than its deposition rate, a thermodynamically stable nanocrystal will be formed. That is, growth occurs based on a thermodynamic regime (a.k.a. the minimum energy principle). In contrast, growth can occur based on a kinetic regime [60, 155–158]. Under thermodynamic control, the produced Pt-based nanocrystals should be covered by facets with the lowest surface energy (the intrinsic surface energy of different facets follows the order of {111} < {100} < {110} < high-index facets; however, the facets can be tuned by structure-directing agents [159]) or that have minimal surface areas. Therefore, spherical nanocrystals or nanocrystals with exposed {111} facets (octahedra, decahedra, or icosahedra) are thermodynamically favorable products. During synthesis, thermodynamically stable Pt-based nanocrystals can be formed at sufficiently high temperatures or with sufficiently long reaction times. In the kinetic growth regime, if the growth rate (i.e., the deposition rate) in the direction perpendicular to a facet is slow, the facet is retained. Conversely, if the growth rate in the direction perpendicular to a facet is fast, the facet is lost. Therefore, seeds without a well-determined morphology will grow into a specific shape [62, 154].

Generally, the final shape is supposed to be a thermodynamically controlled product while the entire growth process is carried out under kinetic control [52]. Various shaped Pt-based electrocatalysts have been successfully prepared by selecting proper soft templating agents or structure capping agents [50]. Various shaped micelles or reverse micelles are formed by tuning the kinds or contents of surfactants or solvents, which can be used as soft templates for the synthesis of shaped Pt-based electrocatalysts. Under the confined function of micelles or reverse micelles, Pt seeds grow into nanocrystals with a certain shape [144, 148]. Structure capping agents include surfactant molecules, polymers, organic solvents, reducing agents, ions (halide ions), ligands of metal precursors, or gas molecules (CO). They selectively adsorb on specific crystal facets since different facets have different atomic arrangements and electronic structures, which change the surface energy of the facet and thus the relative growth rate along different directions [60, 156, 160, 161]. Based on density functional theory (DFT) calculations, the surface energy (*φ*) of the {100} facets is 0.26 eV per atom higher than that of the {111} facets on a clean Pt surface. After coadsorption of amine and CO (0.25 monolayer/0.25 monolayer), *φ*{100} is less than that of the {111} facets by 0.02 eV per atom, indicating that the {100} facets become stabler than the {111} facets [160]. Therefore, the shape-controlled synthesis of Pt-based electrocatalysts can be carried out by changing the metal precursors, capping agents, reducing agents, proper soft templating agents or solvents in a reaction system.

The synthesis strategies for spherical and nonspherical Pt-based electrocatalysts with and without a carbon support are summarized here. Nonspherical Pt-based electrocatalysts include Pt-based polyhedrons, nanocages, nanoframes, and 1D and 2D nanostructures. The impacts of experimental parameters on the size and shape of Pt-based electrocatalysts are reviewed in detail, which is expected to help better control particle size and shape and develop new reaction systems suitable for mass production.

### Synthesis of Spherical Pt-Based Nanoparticles

Spherical Pt-based nanoparticles are often not strictly spherical, especially those prepared by one-pot synthesis methods [162]. Although the total surface free energy of spherical nanoparticles is minimal under vacuum, a spherical morphology is almost impossible in the presence of solvents, reductants, ligands, ions, surfactants, or supports [157]. In general, prepared Pt-based nanoparticles are near-spherical, semispherical (or spherical-crown), ellipsoidal, or semiellipsoidal. The formation of semispherical or semiellipsoidal particles may result from the wetting of Pt-based nanoparticles on carbon, which can enhance metal–support interactions. Hereafter, for convenience, we vaguely call them Pt-based spherical nanoparticles.

Compared with nonspherical morphologies such as nanowires, nanoplates, polyhedrons, nanocages, and nanoframes, the synthesis systems of carbon-supported Pt-based spherical nanoparticles are usually less complex, as some structure-capping agents are not commonly needed, which favors one-pot synthesis [30, 144, 163, 164]. The one-pot and ex situ mixing synthesis methods for preparing spherical carbon-supported Pt-based electrocatalysts are shown in Tables 7 and 8, respectively. Methods include chemical reduction in water, organic solvents (including but not limited to ethylene glycol or benzyl ether), or mixed solutions; electrochemical deposition; and impregnation followed by H_2_ reduction. In comparison with those in aqueous solution, nanoparticles synthesized in organic solvents commonly possess smaller sizes, as shown in Table 7. However, some of the organic solvent molecules adsorbed on the carbon-supported Pt-based electrocatalysts are often difficult to remove, generally needing thermal treatment at elevated temperatures. For example, being treated at 300 °C for 1 h in an Ar atmosphere to remove ethylene glycol (EG) [165] or 260 °C in 20% O_2_/80% N_2_ for 90 min to remove oleylamine and oleic acid [65]; these high temperatures more or less cause an increase in particle size. Reactions in an aqueous solution do not have this problem, which will simplify postsynthesis treatment. In reported synthesis procedures, the size and dispersion of Pt-based spherical nanoparticles on carbon can be controlled by using functionalized carbon supports [88, 103, 151, 166, 167], varying the concentration of precursors or capping agents [65], using ligands (for example, sodium citrate coordinated with Pt, Pd, or Ni ions form new complexes with changed reduction potentials, which is beneficial for forming small, well-dispersed alloys [168, 169]), changing the pH [103, 165, 168, 170, 171] or solvent [143, 172] (adding water into EG can accelerate the reduction of Pt^2+^ by EG [66]), reducing metals confined in reverse micelles, controlling the reaction temperature [30], and varying the reducing capability of the reducing agent [65].

**Table 7:** Summary of the methods for preparing spherical carbon-supported Pt-based nanoparticles by one-pot methods (arranged by the type of solvent)

Catalyst	Precursor	Solvent^a^; reductant	Reaction conditions	Size or dispersion control	Size, loading	Refs.
PtRu/PPDA-MWCNT	H_2_PtCl_6_, RuCl_3_	H_2_O (pH 10); NaBH_4_	90 °C for 2 h	PPDA-MWCNT	3.5 nm; 10.52 wt_Pt_%, 5.26 wt_Ru_%	[88]
Pt/RGO	K_2_PtCl_6_	H_2_O; NaBH_4_	In N_2_	Support	4.6 nm, 20 wt%	[167]
PtM/VXC-72R (M = Fe, Co, Ni)	H_2_PtCl_6_, M(NO_3_)_*x*_	H_2_O; NaBH_4_	Ice water for 3 h	Low reaction temperature	~ 3 nm, 10 wt%	[293]
Pt/NG-TiON	H_2_PtCl_6_	H_2_O (pH 10–11); FA	–	Support, pH	2.02 nm, 33 wt%	[103]
Pd_3_Au@Pt/C	K_2_PdCl_4_, NaAuCl_4_, K_2_PtCl_4_	H_2_O; AA	Stirred for at least 3 h	–	3–6 nm, 19.3 wt%	[164]
Au-doped Pt_75_Co_23_/VXC-72R	Pt_73_Co_27_/VXC-72R, KAuCl_4_	H_2_O; galvanic replacement	0.5 mM Ar-saturated HNO_3_ aq., stirred at 800 rpm (1 rpm = 1 r min^−1^) for 10 min	–	(4.4 ± 1.5) nm, 20.1 wt_Pt_%	[294]
Pt_5_Co/C	Pt/C, Co(NO_3_)_2_	H_2_O/methanol (2/1); NaBH_4_	Dropwise addition of NaBH_4_ methanol solution; annealed at 600 °C for 7 h in H_2_/N_2_; dealloyed in 1 M HNO_3_ at 70 °C for 24 h	–	7–8 nm, ~ 20 wt%	[295]
Pt_1_Co_9_/PG	H_2_PtCl_6_, CoCl_2_	Ethanol/H_2_O (1/3); NaBH_4_	Stirred for 20 min in N_2_	–	8 nm, 30 wt%	[296]
Pt/(FeNC@graphene)	K_2_PtCl_4_	H_2_SO_4_ aq.; electrochemical deposition	Pulse: 0.9 V for 1 s, 0.4 V for 1 s, OCP for 30 s	–	2.0 nm, –	[297]
PdPt/VXC-72R	Na_2_PtCl_6_, PdCl_2_	EG (pH 10–12/9–10); EG	180/140 °C for 6 h in N_2_	Sodium citrate, pH	2.7–2.9 nm, 20 wt%	[168]
PtCu/VXC-72R	H_2_PtCl_6_, CuCl_2_	EG (pH 10); EG	130 °C for 3 h	pH	~ 3 nm, –	[171]
PtRh/VXC-72R	H_2_PtCl_6_, RhCl_3_	EG; EG	In an autoclave, heated at 150 °C for 3 h	–	2.3 nm, 50 wt%	[163]
PtPd/OMC	Na_2_PtCl_6_, PdCl_2_	EG (pH 11); EG	130 °C for 3 h in Ar	pH	2.35 nm, 10 wt%	[165]
Pt/holey graphene	H_2_PtCl_6_	EG:H_2_O (1/1); NaBH_4_	52 °C for 2 h	Holey graphene	–, 22 wt%	[151]
Pt/PANI-graphene	H_2_PtCl_6_	EG/H_2_O; EG	130 °C for 4 h	Viscosity of the solvent, support	~ 3 nm, –	[143]
Pt/Py-MWCNT	H_2_PtCl_6_	EG:H_2_O (3/1); EG	120 °C for 2 h	Py-MWCNT, the viscosity of solvent	~ 3 nm, 1–30 wt%	[172]
PtCo/Py-MWCNT	H_2_PtCl_6_, Co(NO_3_)_2_	EG:H_2_O (3/1) (pH 12); EG	120–130 °C for 7 h	Py-MWCNT, pH	1.6 nm, ~ 27 wt%	[298]
Pt/VXC-72R or EC-600	K_2_PtCl_4_	CH_3_Cl/H_2_O; NaBH_4_	1 600 rpm, room temperature	Reverse micelles adsorbed on carbon	2.6–3.6 nm, 18.6–59.9 wt%	[30]
Pt_3_Pd-CeO_2_/C	H_2_PdCl_4_, K_2_PtCl_6_	–; H_2_	350 °C for 3 h in Ar/H_2_	–	–, ~ 2 wt%	[134]
Pt_3_Co@Pt/C, Pt_3_Co/VXC-72R	H_2_PtCl_6_, CoCl_2_	–; H_2_	150–700 °C for 2–6 h in H_2_/N_2_	–	2.4 nm, –; 5 nm, 40 wt%; 5 nm, –	[299] [300] [301]
N-doped Pt_3_Co/C	H_2_PtCl_6_, CoCl_2_	–; H_2_	300 °C for 2 h in H_2_/N_2_, then 700 °C for 2 h in NH_3_	–	7.9 nm, 19.94 wt%	[302]
Pt_73_Co_27_/VXC-72R	Pt(acac)_2_, Co(acac)_2_	–; H_2_	500 °C for 2 h in H_2_/N_2_, then 500 °C for 5 h in N_2_, etched in 0.5 mM HNO_3_ for 10 min	–	(4.4 ± 1.4) nm, 20.1 wt_Pt_%	[294]
PtM/RGO (M = Fe, Ni, Co)	[M(bpy)_3_]SO_4_, H_2_PtCl_6_	–; –	Adsorption and decomposition at 700 °C for 6 h in Ar	Bipyridine ligands (forming a protective shell of N-doped C)	2–8 nm, 22–46 wt%	[303]

^a^The ratio of mixed solvent is in volume ratio

PPDA: *p*-phenylenediamine; NG-TiON: N-doped graphene-N-doped TiO_2_; FA: formic acid; EG: ethylene glycol; AA: ascorbic acid; PG: nanoporous graphene; OCP: open circuit potential; OMC: ordered mesoporous carbon; PANI: polyaniline; Py: pyridine; bpy: 2,2’-bipyridine

**Table 8:** Summary of the methods for preparing spherical carbon-supported Pt-based nanoparticles by ex situ mixing methods (arranged by the type of solvent)

Catalyst	Precursor	Solvent; reductant	Reaction conditions	Size or dispersion control	Size, loading	Refs.
PtCo/C	Pt(acac)_2_, Co_2_(CO)_8_	BE; HDO	270 °C for 30 min in N_2_	Concentration of precursor, capping agent (OAm/OAc)	2.8, 3.9, 4.6, 7 nm; 37, 24, 40, 36 wt%	[65]
PtNi/C	Pt(acac)_2_, Ni(acac)_2_	BE; Li(C_2_H_5_)_3_BH (for 2 nm)/HDO	100 °C for 20 min in N_2_ (for 2 nm), or 270 °C for 30 min in N_2_	Reducing power of reductant	2.7, 6.1, 6.6, 8.7 nm; 38, 44, 45, 24 wt%	[65]
PtCu/VXC-72R	K_2_PtCl_4_, CuSO_4_	EG; AA	80 °C for 30 min (forms Cu nanoparticles), then 90 min (Pt displaces Cu)	–	4 nm, 20 wt%	[162]
Pt/OMC	H_2_PtCl_6_	EG; EG	150 °C for 4 h in Ar	–	(1.4 ± 0.23) nm, 9.6 wt%	[166]
PtRh/VXC-72R	H_2_PtCl_6_	EG; EG	3 min at 160 °C	NaOH/Pt molar ratio (4.5–10)	2.1 nm (10), 2.9 nm (5.5), 4.0 nm (5), 5.5 nm (4.5); 50 wt%	[304]

HDO: 1,2-hexadecanediol; OAm: oleylamine; OAc: oleic acid; Li(C_2_H_5_)_3_BH: lithium triethylborohydride

### Synthesis of Pt-Based Polyhedrons

Electrocatalytic reactions are structure sensitive, and electrocatalytic activity varies significantly with the arrangements or shapes of the surface atoms of Pt-based electrocatalysts. For example, for pure Pt in HClO_4_ (a nonadsorbing electrolyte), the order of ORR activity of low-index crystal facets is Pt{110} > Pt{111} > Pt{100}, and for Pt_3_Ni, the order is Pt_3_Ni{111} > Pt_3_Ni{110} > Pt_3_Ni{100}. For pure Pt in H_2_SO_4_ (an adsorbing electrolyte), the order is Pt{110} > Pt{100} > Pt{111} due to the stronger adsorption of SO_4_^2−^ or HSO_4_^−^ on Pt{111} facets than other facets, while in KOH (an adsorbing electrolyte), the order is Pt{111} > Pt{110} > Pt{100} due to the different adsorption of OH^−^ on the different crystal facets [170, 173]. However, spherical Pt-based nanoparticles have no precise facets on the surface. In many cases, the performance of Pt-based electrocatalysts is improved by engineering the morphology to expose highly active facets. In general, Pt-based electrocatalysts with polyhedral shapes, such as {100} crystal facet-closed nanocubes (NCs); {111} crystal facet-closed nanotetrahedrons (NTs), nanooctahedrons (NOs), and nanoicosahedrons (NIs); or {100} and {111} crystal facet-closed nanocuboctahedrons (a.k.a. nanotruncated octahedrons (NTOs)), show higher activity and durability than spherical nanoparticles [163, 174, 175]. Moreover, from a geometric point of view, a sphere has a minimum surface area for a certain mass or volume. Regarding electrocatalysts with a specific mass and a given particle size, Pt-based polyhedrons will have a larger surface area than spherical nanoparticles and thus a higher ECSA. Therefore, the effects of the synthesis, size, shape, and composition on the electrocatalytic activity and durability of Pt-based polyhedrons have been widely studied [50, 52].

During the synthesis of Pt-based polyhedrons, several key experimental parameters are responsible for the well-defined shape, high shape selectivity, good dispersion, small size and narrow size distribution due to affecting nucleation and growth. These parameters include the solvents, pH of the reaction solution [176, 177], reductants, structure-directing agents, metal precursors, supports, addition sequence of reactants [174], reaction temperature, temperature ramp rate, and secondary species generated during the reaction. In several reaction systems, a specific parameter can be ascribed to be the key factor affecting size and shape. However, for most cases, the synergistic effect of various parameters in a reaction system is responsible for the well-controlled preparation of polyhedrons [178]. Table 9 summarizes several reported reaction systems for preparing Pt-based NCs, NOs, NTs, NIs, and NTOs supported on carbon by one-pot or ex situ mixing methods and by carbon-free methods. The effects of experimental parameters on the formation of Pt-based polyhedrons in practical cases are summarized as follows.

**Fig. 8:**
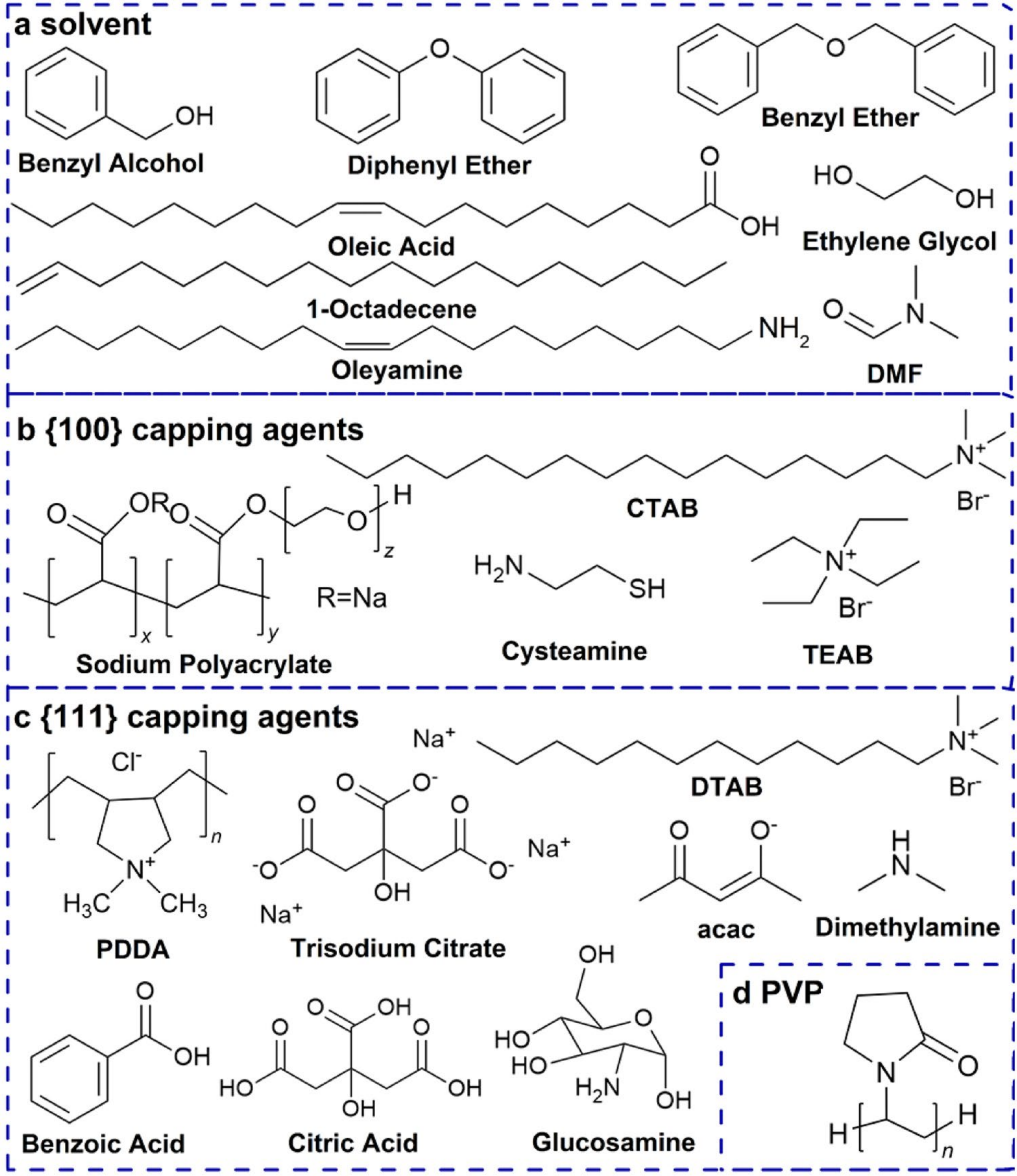
Molecular structure of several commonly used organic materials. **a** Frequently used organic solvents for preparing Pt-based polyhedrons, including benzyl alcohol, ethylene glycol, 1-octadecene, oleic acid, oleylamine, benzyl ether, diphenyl ether, and *N*,*N*-dimethylformamide. **b** Commonly used capping agents for preparing {100} facet-bound Pt-based NCs in the literature, including TEAB, CTAB, cysteamine, and sodium polyacrylate. **c** Commonly used capping agents for preparing {111} facet-bound Pt-based polyhedrons, including benzoic acid, critic acid, PDDA, trisodium citrate, glucosamine, DTAB, dimethylamine, acetylacetonate (acac), TEAB and CTAB (the molecular structures of TEAB and CTAB are shown in (**b**)). **d** PVP, which is usually used as a stabilizer

1. *Solvents*. The types of solvent used or the amounts of different solvents in a mixture are able to affect the selection of structure-capping agents, metal precursors or reducing agents, along with the reaction conditions. Different solvents have different properties, such as different boiling points, polarities, and viscosities, which influence the physicochemical properties of the solute in them, such as its solubility [159], and the reaction conditions, such as the reaction temperature. Commonly used solvents include H_2_O, benzyl alcohol, ethylene glycol, 1-octadecene, oleic acid, oleylamine, benzyl ether, diphenyl ether, *N*,*N*-dimethylformamide or a mixture of two or more (see Table 9 and Fig. 8a for more details). In some specific reaction systems, solvents function as not only solvents but also reducing agents, assistant structure-directing agents, or stabilizers. In some cases, oleylamine, *N*,*N*-dimethylformamide, benzyl alcohol, or ethylene glycol usually function as reducing agents [7, 178–181]. In oleylamine or oleylamine/oleic acid systems, oleylamine can help stabilize Pt_3_Ni{111} facets by lowering their surface energy [179] or adsorb on the surface of Pt_3_Ni NCs to hinder the aggregation of NCs [159]. In addition, the amine group of oleylamine can coordinate with Pt ions, slowing down the reduction rate of Pt ions at the early stage of synthesis to form large polyhedrons due to producing a small number of nuclei [150, 182]. Therefore, the size of Pt polyhedrons can be readily controlled by simply adjusting the amount of oleylamine in oleylamine/oleic acid systems.
2. *Reductants (kind and concentration)*. Generating Pt-based polyhedrons generally requires a slow reducing rate. Thus, a reducing agent with a low reducing capacity should be chosen, which is different from preparing spherical Pt-based electrocatalysts that can use NaBH_4_ or ascorbic acid [183]. Commonly used reducing agents include some organic solvents as introduced above, formaldehyde (HCHO), glucosamine, or poly(vinylpyrrolidone) (PVP, hydroxyl end group contributes to its reducing capability), as shown in Table 9. Since the reducing power of PVP is weak, Pt seeds can slowly grow into octahedra at a low concentration of free Pt^0^ in solution while preventing additional nucleation events from occurring [183]. As the amount of HCHO solution (40 wt%) is increased from 0 to 0.005 and then to 0.010 mL, the reducing rate accelerates, and the shape selectivity of PtPd NIs increases to above 80%. After further increasing the amount of HCHO solution, the obtained nanoparticles gradually change from multiply twinned particles to single-crystalline particles with much smaller sizes. When 0.20 mL of HCHO solution (40 wt%) is employed, PtPd NTs (approximately 5 nm) with a shape selectivity above 80% can be prepared [176].
3. *Structure-directing agents (a.k.a. structure-capping agents or capping agents; kind, molecular weight, alkyl chain lengths, or concentration* [159, 183]*)*. In the synthesis of Pt-based polyhedrons, commonly used structure-capping agents include one or more of the following: CO gas, metal carbonyls (e.g., Fe(CO)_5_, Co_2_(CO)_8_, W(CO)_6_), halides (Cl^−^, Br^−^, I^−^), surfactants, polymers, and other organic molecules with oxygen-, nitrogen-, or sulfur-containing groups (Fig. 8b–d and Table 9). Among them, the capping agents probably used for preparing {100} facet-bound Pt-based NCs include CO gas, CO or W^0^ (supplied by a metal carbonyl), Ag species (supplied by AgNO_3_), Br^−^ (supplied by KBr), tetraethylammonium bromide (TEAB), CTAB, I^−^ (supplied by KI), cysteamine, sodium polyacrylate or PVP (as shown in Fig. 8b, d and Table 9). Commonly used capping agents for synthesizing {111} facet-bound Pt-based NOs, NTs or NIs include CO gas, metal carbonyls, Ag species (supplied by AgNO_3_), TEAB, Cl^−^ (supplied by NaCl), benzoic acid, CTAB, citric acid, poly(diallyldimethylammonium chloride) (PDDA), C_2_O_4_^2−^ (supplied by NaC_2_O_4_), decyltrimethylammonium bromide (DTAB), trisodium citrate, or PVP (as shown in Fig. 8c, d and Table 9). Although some capping agents can stabilize both the {100} and {111} facets, they tend to stabilize one kind of crystal facet in a specific reaction system and at a given concentration. For example, at a relatively low concentration of Br^−^ supplied by TEAB, Br^−^ tends to coordinate with metal precursors, form stable complexes, slow the reduction rate, and generate thermodynamically stable {111} facet-bound NOs. At a high concentration, Br^−^ prefers to selectively adsorb on the {100} facets, forming an NC shape. At a medium concentration, {111} and {100} facet-bound NTOs are formed [159].
4. *Metal precursors (kind or concentration)*. The valence state or ligands of Pt precursors [62, 63] or the addition of non-Pt precursors [176, 178, 182] also play a crucial role in the formation of well-defined Pt-based polyhedrons. If (NH_4_)_2_PtCl_6_ is partially replaced with (NH_4_)_2_PtCl_4_, which is reduced more easily, the number of nuclei formed at the initial stage can be increased, resulting in a smaller size [62]. In the synthesis of PtNi NOs, the precursor ligand acetylacetonate (Ni(acac)_2_ and Pt(acac)_2_) is regarded as critically influencing the formation and size control of PtNi NIs by modifying the metal redox potential and reduction rate [63]. Moreover, the addition of a non-Pt precursor also affects the resulting shape or size due to different standard reduction potentials, different reduction kinetics, and different adsorption properties of the structure-capping agent on non-Pt metal from Pt, finally forming different alloy shapes from pure Pt. For example, in the absence of a Pd precursor, only uncontrollable Pt nanocrystals are formed. When adding a Pd precursor, well-defined PtPd NCs or NOs are obtained due to the coreduction of PtPd nanocrystals induced by Pd nuclei formed at the initial stage [178]. It was found that the adsorption of CO on Pt{100} facets is much stronger than on Pt{111} facets, favoring the formation of Pt NCs. When a sufficient amount of second metal, e.g., Ni, is introduced, the absorption preference of CO is tuned from the {100} facets to the {111} facets due to the stronger adsorption of CO on the Ni{111} facets than on the Ni{100} facets, eventually forming PtNi NOs [182].
5. *Support*. Although in some cases the presence of carbon disturbs shape control [30, 144], it has been found that carbon supports can sometimes also be used to promote the formation of Pt-based polyhedrons. Functional groups on the surface of carbon act as binding sites for anchoring nanoparticles, thereby inducing anisotropic growth [180, 184–186].
6. *Reaction temperature and temperature ramp rate*. The reaction temperature can affect the size and shape of prepared Pt-based electrocatalysts by changing the nucleation and growth rate. When Fe(CO)_5_ is injected into the reaction solution at 180 °C, the nucleation rate is fast, and most of the Pt precursors are consumed during nucleation, resulting in small polyhedral Pt (~ 3 nm) without preferential exposure of the specific facet due to insufficient feedstock for growth. If Fe(CO)_5_ is injected at 120 °C, a smaller number of Pt nuclei are formed, and these Pt nuclei can grow into NCs under the control of a capping agent with sufficient feedstock. If Fe(CO)_5_ is injected at 160 °C between the above temperatures, a truncated cubic shape is obtained [68]. In addition, during heating, the ramp rate also influences the size of Pt-based electrocatalysts. Regarding the synthesis of PtNi NOs/C by impregnation and then reduction in CO and H_2_ gas at 200 °C, the average edge size of the produced octahedral Pt_3_Ni/C monotonically increases from (4.5 ± 1.3) to (6.0 ± 1.4), (6.8 ± 1.5), and (8.1 ± 1.6) nm by using different ramp rates of 10, 5, 2, and 1 °C min^–1^, respectively [69].
7. *Secondary species*. Secondary species are not the matter added before the reaction but are generated from the initially added matter during the reaction. New metal complexes are formed because the initial precursor ligands are replaced by solvent or capping agent molecules (as ligands), leading to a low nucleation/growth rate [150, 176, 182, 187], or new capping agents are produced by the reaction between the initially added matter. For example, if ethylene glycol and *N*,*N*-dimethylformamide are simultaneously used in the reaction, dimethylamine is yielded in situ, which has a high affinity for Pt{111} facets and promotes the formation of Pt NTs [185].

**Table 9:** Summary of the methods for preparing Pt-based polyhedrons supported on carbon by one-pot (OP) or ex situ mixing (EM) methods and by carbon-free methods (arranged by the type of solvent)

Shape	Catalyst	Precursor	Solvent^a^; reductant	Capping agent; stabilizer^b^	Reaction conditions	Selectivity, edge length, loading	Refs.
NCs (OP)	Pt/C	Pt(acac)_2_	BA; BA	CO (1 bar, 1 bar = 100 kPa); –	180 °C for 3 h	–, 2–3 nm, –	[180]
NCs (EM)	PtNi/C	Pt(acac)_2_, Ni(acac)_2_	BA; BA	CO, Br^−^ (KBr); PVP	150 °C for 4 h under CO	95%, (16.1 ± 1.7) nm, 10 wt%	[260]
NOs (EM)	PtNi/C	Pt(acac)_2_, Ni(acac)_2_	BA; BA	BA; PVP	In autoclave, 150 °C for 12 h	95%, 11.8 nm, 10 wt%	[260]
NTOs (EM)	PtNi/C	Pt(acac)_2_, Ni(acac)_2_	BA; BA	Aniline; PVP	In autoclave, 150 °C for 12 h	–, 16–4.8 nm by varying the amount of aniline, 10 wt%	[260]
NCs (OP)	Pt/C	H_2_PtCl_6_	EG/H_2_O (3/1); EG	Br^–^ (KBr, Br^–^:Pt = 300); –	150 °C for 3 h, addition of 30% H_2_PtCl_6_, after 15 min addition of KBr, after 20 min addition of 70% H_2_PtCl_6_	–, 6–8 nm, –; –, 9 nm, –; –, 8 nm, 20 wt%	[174, 305, 306]
NOs (EM)	PtIr/C	K_2_PtCl_4_, Ir(acac)_2_	EG; EG	Br^–^ (TEAB, 0.6 mM); PVP	In autoclave, 180 °C for 24 h	–, (4.2 ± 0.4) nm, –	[262]
NTOs (EM)	PtIr/C	K_2_PtCl_4_, Ir(acac)_2_	EG; EG	Br^–^ (TEAB, 1.8 mM); PVP	In autoclave, at 180 °C for 24 h	–, (4.0 ± 0.3) nm, –	[262]
NCs (EM)	PtIr/C	K_2_PtCl_4_, Ir(acac)_2_	EG; EG	Br^–^ (TEAB, 3 mM); PVP	In autoclave, 180 °C for 24 h	–, (5.8 ± 0.4) nm, –	[262]
NCs	Pt	(NH_4_)_2_PtCl_6_, (NH_4_)_2_PtCl_4_ (1/4, 1/1, 4/1)	EG; EG	Br^–^ (TEAB); PVP	Increasing to 180 °C at 60 °C min^–1^ and then kept for 20 min in Ar	> 80%; (6.9 ± 1.8) nm (1/4), (5.9 ± 0.7) nm (1/1), (5.0 ± 0.4) nm (4/1); –	[62]
	Pt	H_2_PtCl_6_	EG; EG	PVP; –	In boiling EG, addition of a mixture of PVP and H_2_PtCl_6_ over 16 min	–, (6.1 ± 1.3) nm, –	[187]
	Pt	H_2_PtCl_6_	EG; EG	Ag species (AgNO_3_, Ag/Pt = 1.1); PVP	In boiling EG, addition of PVP and then H_2_PtCl_6_ over 16 min	~ 80%, (9.4 ± 0.6) nm, –	[181]
NTOs	Pt	H_2_PtCl_6_	EG; EG	Ag species (AgNO_3_, Ag/Pt = 11); PVP	In boiling EG, addition of PVP and then H_2_PtCl_6_ over 16 min	~ 100%, (9.1 ± 0.6) nm, –	[181]
NOs	Pt	H_2_PtCl_6_	EG; EG	Ag species (AgNO_3_, Ag/Pt = 32); PVP	In boiling EG, addition of PVP and then H_2_PtCl_6_ over 16 min	~ 80%, (9.8 ± 0.6) nm, –	[181]
NOs (OP)	PtNi/C	Pt(acac)_2_, Ni(acac)_2_	EG; EG	PDDA; –	170 °C for 2 h in N_2_	–, 10–15 nm, ~ 27 wt%	[307]
NCs (OP)	Pt/C	Pt(acac)_2_	ODE/OAm/OAc (8/1/1); OAm	Fe(CO)_5_; –	Addition of Fe(CO)_5_ at 160 °C, then heated at 200 °C for 1 h in Ar	–, 5–6 nm, 50 wt%	[8]
NCs	Pt, Pt_3_Co, Pt_3_Fe	Pt(acac)_2_, FeCl_2_, (CH_3_CO_2_)_2_Co	OAm/OAc (4/1); OAm	W^0^ (W(CO)_6_); OAm	Addition of W(CO)_6_ at 130 °C, and then in Ar at 240 °C for 30–60 min	–; (8.1 ± 0.6) nm (Pt), (9.1 ± 0.5) nm (Pt_3_Co), (7.9 ± 0.6 )nm (Pt_3_Fe); –	[159]
	Pt_3_Ni	Pt(acac)_2_, NiCl_2_	OAm/OAc (4/1); OAm	W^0^ (W(CO)_6_); OAm	Addition of W(CO)_6_, NiCl_2_ at 130 °C, kept at 240 °C for 15 min in Ar	–, (8.1 ± 0.6) nm, – –, (10.3 ± 0.3) nm, –	[159] [179]
	Pt	Pt(acac)_2_	OAm/OAc (4/1); OAm	CO; OAm	180 °C in a CO flow (~ 30 mL min^–1^) for 40–60 min	–, 10.3 nm, –	[160]
	Pt	Pt(acac)_2_	OAm/OAc (1/1); OAm	Fe(CO)_5_; –	Addition of Fe(CO)_5_ at 120 °C, then heated at 200 °C for 1 h in Ar	–, 7 nm, –	[68]
	Pt	Pt(acac)_2_	BE/OAm/OAc; CO	–	200 °C for 15 min	–, 8.9 nm, –	[308]
	PtRu	Pt(acac)_2_, Ru(acac)_3_	OAm; W(CO)_6_	Br^–^ (CTAB); –	In autoclave, 185 °C for 5 h in N_2_	~ 100%, 6.2 nm, –	[218]
NOs (EM)	PtNi/C	Pt(acac)_2_, Ni(acac)_2_	BE, OAm/OAc; OAm	CO (W(CO)_6_); OAm/OAc	Addition of W(CO)_6_ at 130 °C, then kept at 230 °C for 40 min	–; 6, 9, 12 nm (OAm/OAc 1/4, 2/1, 6/1); 25 wt%	[150, 182]
	PtNiRh, (PtNi)/C	Pt(acac)_2_, Ni(acac)_2_, Rh(acac)_3_	OAm/OAc (3/2, 4/1); OAm, W(CO)_6_	CO (W(CO)_6_); –	Addition of W(CO)_6_ at 130 °C, then kept at 230 °C for 40 min	–, ~ 7.9 nm, 31 wt%	[309, 261]
	PtNi, (PtCo)/C	Pt(acac)_2_, Ni(acac)_2_, Co(acac)_2_	OAm/DPE (9/1); CO	CO; DPE	210 °C for 30 min in CO (190 cm^3^ min^–1^)	–, 5 nm, 20 wt%	[310]
NIs (EM)	PtNi, (PtAu)/C	Pt(acac)_2_, Ni(acac)_2_, HAuCl_4_	OAm/OAc (9/1); CO	CO; –	210 °C for 30 min in CO (190 cm^3^ min^–1^)	–; ~ 13 nm (PtNi), ~ 11 nm (PtAu); –	[311]
	PtPd/C	Pt(acac)_2_, Pd(acac)_2_	OAm/DPE (9/1); CO	CO; DPE	210 °C for 30 min in CO (190 cm^3^ min^–1^)	–	[311]
NIs	PdCuPt	Pd(acac)_2_, Pt(acac)_2_, Cu(acac)_2_	OAm/DMF (1/1); –	DTAB; trisodium citrate	In autoclave, 180 °C for 9, 12, 18 or 24 h	–; 6.95, 7.41, 7.87, 8.31 nm (9, 12, 18, 24 h); –	[152]
NTOs	Pt	Pt(acac)_2_	OAm/OAc (1/1); OAm	Fe(CO)_5_; –	Addition of Fe(CO)_5_ at 160 °C, then heated at 200 °C for 1 h in Ar	–, 5 nm, –	[68]
NCs	PtPd	K_2_PtCl_4_, Na_2_PdCl_4_	DMF; DMF	I^–^ (75 mg NaI); PVP	130 °C for 5 h	~ 100%, 7.2 nm, –	[178]
NIs (OP)	PtNi, PtNiCo/C	Pt(acac)_2_, Ni(acac)_2_	DMF; DMF	Benzoic acid; –	160 °C for 12 h	–; ~ 4.2 or 6 nm (PtNi), 4.4 nm (PtNiCo); –	[7, 22, 255]
	PtNi/GC (CNTs)	Pt(acac)_2_, Ni(acac)_2_	DMF; DMF	CTAB; –	In autoclave, 160 °C for 12 h	–; 5–7 nm (GC), 12 nm (CNTs); ~ 40 wt%	[67, 312, 313]
	PtNiCo/C or PtNi/C	Pt(acac)_2_, Ni(acac)_2_, Co_2_(CO)_8_, Co(acac)_2_	DMF; citric acid	Citric acid, CO (Co_2_(CO)_8_ or W(CO)_6_); –	165 °C for 24 h	–, (5.6 ± 0.6) nm, –	[186]
NIs (EM)	PtNi/C	Pt(acac)_2_, Ni(acac)_2_	DMF; DMF	Acac; –	In autoclave, increased to 120 °C within 10 min, and then kept for 16, 28 or 42 h	–; 9, 9.2, 9.5 nm (16, 28, 42 h); –	[63]
	PdPt/C	H_2_PtCl_6_, Na_2_PdCl_4_	DMF/H_2_O; DMF	Benzoic acid; PVP	130 °C for 5 h	–, (4.3 ± 0.3) nm, –	[85]
NOs, NTs	PtPd	K_2_PtCl_4_, Na_2_PdCl_4_	DMF; DMF	Cl^–^ (NaCl); PVP	130 °C for 5 h	–, 5.3 nm, –	[178]
NTs (OP)	Pt/C	H_2_PtCl_6_	DMF/EG (6/4); DMF	Dimethylamine (DMF); –	In autoclave, 170 °C for 8 h	75%, (7.1 ± 0.9) nm, 28 wt%	[185]
NCs	Pt, Pt/C (EM)	H_2_PtCl_6_	Microemulsion; NaBH_4_	HCl or H_2_SO_4_ (25 wt% in H_2_O); –	Few minutes, room temperature	–, 6–14 nm, –	[177, 314–316]
	Pt	K_2_PtCl_4_	H_2_O; H_2_	SPA; –	12 h	–, 4 to 18 nm, –	[317]
NOs (EM)	Pt/C	Na_2_PtCl_6_	H_2_O; PVP (MW 10–360 kDa, 1 kDa = 1 000 g mol^−1^)	PVP; –	90 °C for 48 h	80%; 5 nm (100 °C, 40 kDa), 6, 9, 15 nm (90 °C, 10, 40, 360 kDa); 15 wt%	[183]
NTs	PtPd	K_2_PtCl_4_, Na_2_PdCl_4_	H_2_O (pH 4, HCl); HCHO (0.4 mL)	C_2_O_4_^2–^ (Na_2_C_2_O_4_); PVP	In autoclave, 180 °C for 2 h	70%, (4.9 ± 0.5) nm, –	[318]
NIs	PtPd	K_2_PtCl_4_, Na_2_PdCl_4_	H_2_O (pH 4, HCl); HCHO (0.01 mL)	C_2_O_4_^2–^ (Na_2_C_2_O_4_); PVP	In autoclave, 180 °C for 2 h	82%, (11.2 ± 0.8) nm, –	[176]
NIs (EM)	Pt or PtM/C	H_2_PtCl_6_, MCl_2_ (M = Ni, Co, Fe, Cu)	H_2_O; glucosamine	Glucosamine; –	In autoclave, 180 °C for 6 h	–, (20 ± 1) nm (PtNi), –	[319]
NCs (OP)	Pt/C	Pt(acac)_2_	ODE; –	Cysteamine; –	120 °C for 20 min, then increased to 230 °C (3–4 °C min^–1^), and kept at 230 °C for 30 min in N_2_	78%, (7.9 ± 1.8) nm, 20.2 wt%	[251]
NCs (OP)	Pt/SiO_2_, Pt/C	Pt(acac)_2_	–; H_2_, CO	CO; –	CO/H_2_ (25/5 mL min^–1^) at 200 °C for 1 h	87%, (8.4 ± 1.9) nm, –	[320]
NOs (OP)	PtCuNi, (PtNi)/C	Pt(acac)_2_, Ni(acac)_2_, Cu(acac)_2_	–; H_2_	CO; –	Increased to 200/210 °C at 1–15 °C min^–1^, then kept for 1 h in H_2_/CO (5/120 (or 5/100) mL min^–1^)	80%, 4–9 nm, 20 wt%	[69, 321–323]

^a^The ratio of mixed solvent is in volume ratio

^b^Stabilizers are favorable for protecting nanoparticles from aggregation and even enhancing shape-controlled synthesis due to the steric effect induced by their large molecular size or possible capping effect

BA: benzyl alcohol; EG: ethylene glycol; ODE: 1-octadecene; OAc: oleic acid; OAm: oleylamine; BE: benzyl ether; DPE: diphenyl ether; DMF: *N*,*N*-dimethylformamide; SPA: sodium polyacrylate

By deliberately controlling the different reaction parameters described above in experiments, many well-defined Pt-based polyhedrons with controlled shapes and compositions and high activity and durability have been successfully prepared. However, most Pt-based polyhedrons are large, as shown in Table 9, resulting in a low Pt utilization efficiency. Methods that synthesize small, well-defined Pt-based polyhedrons (2–5 nm) are still lacking. Additionally, the yield of Pt-based polyhedrons in one batch is still low, commonly dozens of milligrams or even only several milligrams in some cases. Additionally, when scaling up the reaction system to increase the yield, the prepared Pt-based polyhedrons commonly lose their controlled size and shape, resulting in low activity. Therefore, more attention needs to be given to the mass production of well-defined Pt-based polyhedrons.

### Synthesis of Open Pt-Based Nanostructures

Solid Pt-based nanoparticles have a mass proportion of Pt atoms in the interior versus at the surface, especially Pt-based polyhedrons, which are usually large. Because they are blocked by surface atoms and cannot directly participate in the electrocatalytic reaction, the interior Pt atoms of a solid nanoparticle are nearly wasted, which limits their utilization efficiency (the utilization efficiency of Pt atoms refers to the fraction of Pt atoms participating in the reaction relative to the total Pt atoms in the electrocatalyst, which is directly proportional to the ECSA) [188]. The construction of open nanostructures, such as nanocages (NCgs) with porous walls and nanoframes (NFs) composed of only ridges, can not only decrease the consumption of Pt but also improve electrocatalytic activity because the external surface and internal surface can simultaneously participate in the electrocatalytic reaction [21, 189]. Unlike solid nanoparticles, as long as the wall thickness of NCgs or the ridge diameter of NFs are kept within an ultrathin regime, NCgs and NFs can be large while hardly sacrificing their high ECSA and possibly improving their stability.

Wrapping up recent developments, the synthesis of NCgs and NFs mainly involves two stages: the formation of solid nanoparticles and the hollowing out of interior atoms or both interior and side-face atoms. Solid nanoparticles are used as a sacrificial template for preparing NCgs or NFs, and hollowing out interior atoms or both interior and side-face atoms can be carried out through galvanic replacement reactions or chemical etching accompanied by the Kirkendall effect. The Kirkendall effect is a vacancy-mediated mechanism based on the observation of the out-diffusion of atoms inside the solid particle with vacancies diffusing inward and condensing in the interior of the particle, forming a hollow structure [190].

#### Nanocages

##### Galvanic Replacement Reaction

**Fig. 9:**
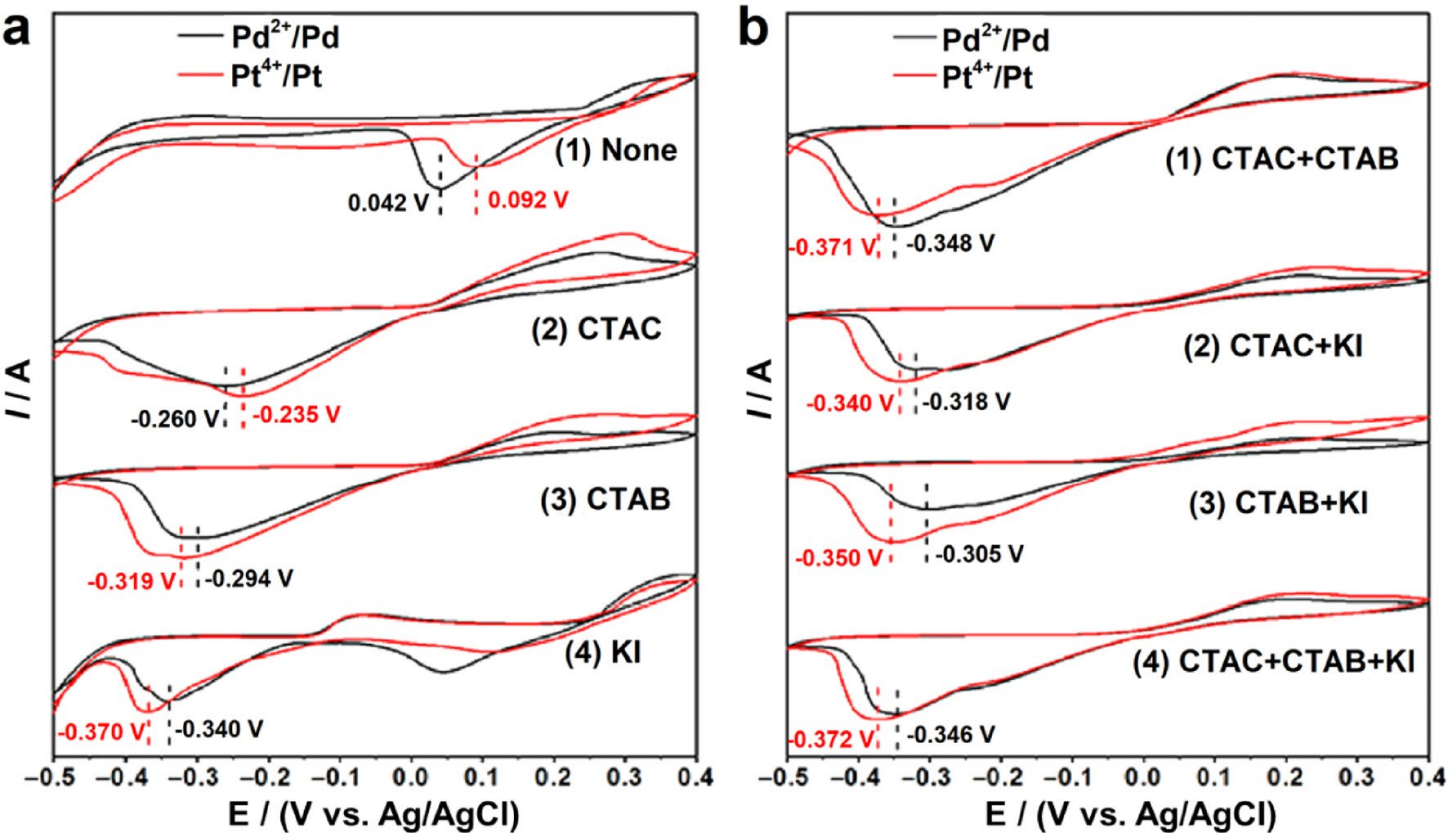
CV curves of the Ag wire electrode in 0.24 mM H_2_PdCl_4_ (black) and 0.24 mM H_2_PtCl_6_ (red) solutions with different additives (reprinted with permission from Ref. [194], copyright © 2017, Springer): **a** (1) none, (2) 25 mM cetyltrimethylammonium chloride (CTAC), (3) 25 mM CTAB, (4) 1.5 mM KI, **b** (1) 25 mM CTAC + 25 mM CTAB, (2) 25 mM CTAC + 1.5 mM KI, (3) 25 mM CTAB + 1.5 mM KI, and (4) 25 mM CTAC + 25 mM CTAB + 1.5 mM KI (counter electrode: Pt mesh; reference electrode: Ag/AgCl; and the scan rate: 20 mV s^–1^)

Regarding the galvanic replacement reaction, a sacrificial metallic template, whose reduction potential should be lower than Pt, is oxidized and dissolved, while Pt ions are reduced and deposited onto the template, which is driven by the discrepancy in the reduction potential between the active metal species and Pt species [49, 190, 191]. As the galvanic replacement reaction continues, surface template atoms are dissolved, interior template atoms diffuse outward, and vacancies diffuse inward, i.e., the Kirkendall process occurs. The sacrificial templates can be pre-prepared first and then added to a reaction solution containing Pt ions, such as Pd, Ag, or Cu nanoparticles (as shown in Table 10), or formed in situ in a reaction solution (as shown in Table 11). Given that the standard reduction potentials for Pd^2+^/Pd (0.951 V vs. SHE), Ni^2+^/Ni (− 0.257 V vs. SHE), or Cu^2+^/Cu (0.341 9 V vs. SHE) are typically more negative than Pt^2+^/Pt (1.18 V vs. SHE) if they are in the same coordination environment, it is unexpected to first form dense Pd, Ni or Cu nanoparticles by simply mixing the Pd, Ni, or Cu precursor and Pt precursor with the same ligands [192]. However, the reduction kinetics or reaction potential of the metal ions can be modulated by adding a sufficient amount of proper ligands, such as halide ions or some N-containing organic molecules (as shown in Tables 11 and 13), because of the different stabilities of the newly formed metal complexes [64, 193, 194]. As a result, Pd, Ni or Cu ions are reduced before the Pt ions in the presence of suitable reducing agents. After the galvanic replacement of Pd, Ni, or Cu nanoparticles with Pt ions, forms Pt-based NCs are finally formed. For example, in the presence of I^–^, Pd(acac)_2_ turns to PdI_4_^2–^, which is more favorable for reduction by *N*,*N*-dimethylformamide than Pt(acac)_2_ [64]. Based on the cyclic voltammetry (CV) results, Pd^2+^ species show a more positive reduction potential than Pt^2+^ species in the presence of I^–^ (KI) or Br^–^ (CTAB), leading to the preferential reduction of Pd^2+^, as shown in Fig. 9 [194]. Similar results can be found in Lou’s work, and their study suggests that the presence of Br^–^ (CTAB) helps reduce Cu^2+^ before Pt^2+^, likely due to their different reduction rates [193].

**Table 10:** Summary of the methods for obtaining nanocages (NCgs) by initially preparing templates for the galvanic replacement reaction (EM: ex situ mixing) (arranged by electrocatalysts)

Nanocage	Template	Reaction system	Wall thickness (size)	Refs.
Cubic Pt NCgs	Ag cubes	Addition of Pt(acac)_4_^4+^ aq. (mixing H_2_PtCl_6_ and acac) at 45 mL h^–1^ and 120 °C for 1.5 h, then washed with saturated NaCl, Fe(NO_3_)_3_ aq. to remove Ag and AgCl	–	[324]
PtAg NCgs	Ag cubes	K_2_PtCl_4_, HCl, and PVP aq. heated at 100 °C, then washed with saturated NaCl, Fe(NO_3_)_3_ aq. to remove Ag and AgCl	10 nm (70 nm, nanoboxes), 1.7 nm (90 nm, popcorns)	[325]
Cubic PtAg NCgs/C (EM)	Ag cubes	Addition of HCl, PVP aq., and then K_2_PtCl_4_ aq. at 1 mL min^–1^ and kept at 40 °C	3.1 nm ((15.8 ± 0.4) nm)	[326]
Spherical Pt_3.78_Co/C (EM)	Co sphere	H_2_PtCl_6_, PVP, HCl aq. (pH = 5) at 70 °C for 2 h in Ar, then treated in 1 M H_2_SO_4_ at 70 °C for 8 h	3.7 nm	[327]
Octahedral PtCu NCgs/C (EM)	Cu-rich PtCu octahedrons	Pt(acac)_2_ and DMF in an autoclave at 115 °C for 5 h	~ 2.1 nm ((23.6 ± 0.8) nm)	[197]
Cubic or octahedral PtPd NCgs/C (EM)	Pd cubes or octahedrons	K_2_PtCl_4_ and CTAC aq. heated at 100 °C for 24 h	∼1.5 nm	[328]

CTAC: cetyltrimethylammonium chloride

**Table 11:** Summarized methods for obtaining nanocages (NCgs) by forming templates in situ for the galvanic replacement reaction (OP: one pot) (arranged by electrocatalysts)

Nanocage	Reaction system	Control of reduction kinetics	Wall thickness (size, selectivity)	Refs.
Spherical PtCo NCgs	Dropwise addition of NaBH_4_ into CoCl_2_ and PVP aq. purged with Ar gas, and then the dropwise addition of K_2_PtCl_6_ aq.	–	– (10–50 nm, 70%–90%)	[329]
Cubic PtCu NCgs	H_2_PtCl_6_, Cu(acac)_2_, CTAB, OAm in an autoclave at 170 °C for 24 h	CTAB	– (~ 20 nm, > 95%)	[193]
PtNi NCgs/RGO (OP)	K_2_PtCl_4_, nickel acetate, GO, NaBH_4_ aq., 10 min of sonication	–	– (~ 10 nm)	[330]
PtPd NCgs	H_2_PdCl_4_, H_2_PtCl_6_, AgNO_3_, CTAB, CTAC, KI, and AA aq. in an autoclave at 140 °C for 12 h	I^–^ (KI), Br^–^ (CTAB), reaction temperature	– (~ 31.5 nm)	[194]
PtPd NCgs	Pt seeds (3.6 nm), H_2_PtCl_6_, H_2_PdCl_4_, CTAC, and AA aq. (pH 3.15) and aged at 30 °C for 3 days	Cl^–^, amount of Pd^2+^, AA, pH	~ 15 nm (~ 42.1 nm)	[195]
Cubic PtPd NCgs	Pd(acac)_2_, Pt(acac)_2_, PVP, NaI in DMF and placed in an autoclave at 140 °C for 12 h	I^–^ (NaI), precursor, reductant, and reaction temperature	– (~ 12.5 nm)	[64]

In addition to ligands, the selection of metal precursors, reducing agents, and temperatures is also important for giving priority to non-Pt-ion reduction [64, 194, 195]. If PtCl_2_ and PdCl_2_ are used as precursors instead of Pt(acac)_2_ and Pd(acac)_2_, only solid PtPd alloy NCgs are obtained even in the presence of I^–^ [64]. When the reducing agent has a strong reducing capability (e.g., ascorbic acid instead of *N*,*N*-dimethylformamide), coreduction occurs, thus forming alloys [64]. If the reaction temperature is low, the diffusion rate of atoms is low and needs a long reaction time to form NCgs. However, a high reaction temperature enhances the reducing rate of all metal ions, similar to the case of using a strong reducing agent [64]. Therefore, the combined influence of ligands, metal precursors, reducing agents, and an appropriate temperature window contributes to forming hollow nanostructures.

##### Chemical Etching

**Fig. 10:**
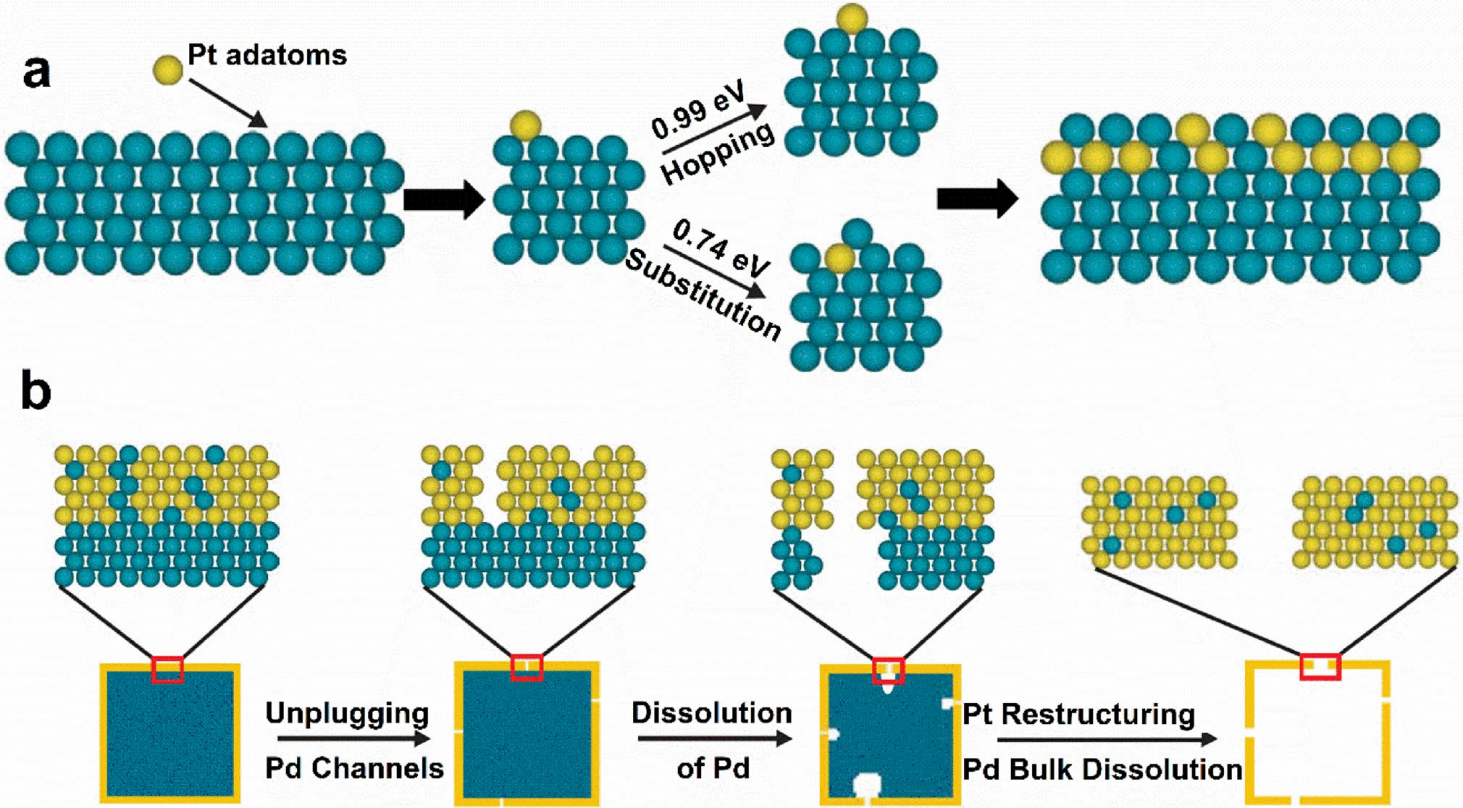
Schematic showing the deposition of the Pt shell and the formation of Pt-based NCgs by etching Pd. **a** Formation of a mixed outer layer structure due to the diffusion of Pt atoms on and along the surface of the Pd shell surface (the so-called hopping mechanism) or substitution of the Pt atoms into the Pd surface (the so-called substitution mechanism). The indicated activation energy barriers are obtained from DFT calculations. **b** Formation of cubic Pt-based NCgs by etching Pd atoms in a Pd@Pt cube. Reprinted with permission from Ref. [189]. Copyright © 2015, the American Association for the Advancement of Science

Pt-based NCgs can also be synthesized by etching non-Pt metal in Pt-based alloys or core@Pt-shell nanocrystals (a.k.a. precursors of NCgs), as shown in Table 12. When a PtNi alloy is used as a precursor, Ni atoms on the surface are dissolved, and the interior Pt and Ni atoms diffuse outward, finally forming Pt-based NCgs [77, 196]. Regarding core@Pt-shell nanocrystals as precursors, the core can include a non-Pt core, Pt-poor core or SiO_2_. If the Pt shell is porous, the etchant can contact the core and easily etch it, forming nanocages with porous walls [197, 198]. If the Pt shell has a flat surface, etching is initiated by dissolving a non-Pt metal such as Pd on the outermost surface of the shell, leaving surface vacancies. The Pd atoms on the surface are probably formed by the intermixing or coreduction of some Pd atoms into the Pt shell during Pt deposition (Fig. 10a). Then, underlying Pd atoms diffuse to the surface vacancies and are etched away, gradually generating channels in the shell that are an atom wide. The Pd atoms in the core can diffuse outward through these channels, forming several voids in the core. When the channels grow to a certain size over time, the direct corrosion of the Pd core begins to occur (Fig. 10b) [189]. The wall of the Pt-based nanocages obtained through this method is always thicker than the Pt shell before etching in TEM images due to interdiffusion and alloying between Pt and Pd, the formation of small holes in the side faces, and the migration of atoms from faces to edges [189, 199, 200].

**Table 12:** Summary of the methods for preparing NCgs by chemical etching (arranged by etching method)

Nanocage	Precursor	Etching methods	Wall thickness (size, loading)	Refs.
Bunched Pt_81_Ni_19_ NCg/C (EM)	Bunched Pt_1.5_Ni nanospheres	0.5 M HNO_3_ aq., 60 °C	~ 2.2 nm (–, 20 wt%)	[196]
Cubic PtPd NCg/RGO (OP)	Pd@Pt cube/RGO	Concentrated HNO_3_, room temperature for 1–5 days	~ 1.7 nm (–)	[201]
PtPdRh MONCgs	PtPdRh@Pt MOs		–	[331]
Icosahedral Pt-rich NCg/C (EM)	Pd@Pt icosahedrons		~ 1 nm (~ 17.7 nm, 20 wt%)	[332]
Porous hollow PtNi/C (OP)	PtNi/C	1 M H_2_SO_4_ aq., (298 ± 5) K for 22 h	1.9–4.3 nm (10–30 nm, 16–24 wt_Pt_%)	[77]
PtPd NCgs or nanorings	Pd@Pt nanoplates	KBr, PVP, FeCl_3_ and HCl aq., 80–100 °C for 1–4 h	1.1 nm (NCs), 1.8 nm (nanorings) (–)	[200]
Icosahedral PtPd NCg/C (EM)	Pd@Pt icosahedrons		1.3 nm (~ 14 nm, 10 wt%)	[199]
Cubic or octahedral PtPd NCg/C (EM)	Pd@Pt cubes or octahedrons		~ 1 nm (20.2 nm cubic NCgs, –)	[189]
Pt MNCgs	SiO_2_@Pt	10 wt% HF for 12 h	20 nm (~ 200 nm, –)	[198]
Porous PtFe NCgs	SiO_2_@PtFe	3 M NaOH aq., 80 °C for 1 h	~ 10 nm (–)	[333]

EM: ex situ mixing; OP: one pot; MONCgs: mesoporous octahedral nanocages; MNCgs: mesoporous nanocages

Chemical etching primarily refers to oxidative etching. Etchants for non-Pt metal include H^+^ from HNO_3_ or H_2_SO_4_ [77, 196, 197, 201], Fe^3+^ [199, 200], CO [202], or dissolved O_2_ [21]. To facilitate the oxidative etching of non-Pt metal, proper coordination ligands such as halide ions or N-containing organic molecules are usually added into the etching solution to stabilize dissolved metal ions or reduce the reduction potentials of metal ions. For example, when Fe^3+^ is used to etch a Pd core, Br^–^ is added to stabilize Pd^2+^ by forming PdBr_4_^2–^ $\left( {{\text{Pd}} + 2{\text{Fe}}^{{3 + }} + 4{\text{Br}}^{ - } \mathop \to \limits^{\Delta } {\text{PdBr}}_{4}^{{2 - }} + 2{\text{Fe}}^{{2 + }} } \right)$, and the standard reduction potential of the PdBr_4_^2–^/Pd pair (0.49 V vs. SHE) is much lower than that of Pd^2+^/Pd pair (0.915 V vs. SHE), thereby facilitating the etching rate of Pd [199]. In addition, if Fe^3+^ is used as an etchant, an acid, such as HCl, should be added to prevent hydrolysis of the Fe^3+^ and Fe^2+^ ions [199]. In the presence of dissolved oxygen, Ni atoms are easier to oxidize than Pt atoms. Oxidized Ni turns into soluble Ni complexes with oleylamine, enhancing the dissolution rate of Ni [21]. Moreover, the strength of oxidative etching plays a critical role in the etching rate and final shape, which is controlled by the reduction potential. Based on the Nernst equation, reduction potentials highly depend on the pH or ligand complexation. Because PdBr_4_^2−^/Pd has a lower reduction potential than PdCl_4_^2−^/Pd (0.59 V vs. SHE), the etching rate of Pd is accelerated by the introduction of Br^–^ instead of Cl^−^ into the etching solution [191]. Compared with that in alkaline media, metals are more easily oxidized by O_2_ in acidic media because the standard reduction reaction of O_2_ in acidic media (1.229 V vs. SHE) is more positive than that in alkaline media (0.401 V vs. SHE).

To maximize the utilization efficiency of Pt atoms, the wall of NCgs should be porous to ensure that reactants and products are able to move in and out of the NCgs and contact the inner surface; moreover, the wall thickness should be as thin as possible on the premise of shape stability.

#### Nanoframes

Compared with NCgs, NFs have a highly open architecture that enhances mass transfer. Similar to the synthesis of NCgs, the synthesis methods for NFs also include galvanic replacement reactions and chemical etching, which are described below.

##### Galvanic Replacement Reaction

As shown in Table 13, the sacrificial templates for preparing NFs are primarily non-Pt metal polyhedrons formed in situ in the reaction solution. Similar to galvanic replacement methods for preparing NCgs, the preferential reduction of non-Pt metal should be carried out by controlling the reduction rates or reduction potential of Pt and non-Pt species [203–207]. However, unlike NCgs, reduced Pt atoms do not uniformly deposit on the template surface; instead, they selectively deposit on high surface energy regions such as vertexes and edges rather than the facets of Pt-based polyhedrons [205, 207]. Therefore, the diffusion of Pt atoms on side facets needs to be hindered.

**Table 13:** Summary of the methods for preparing Pt-based nanoframes (NFs) by the galvanic replacement reaction (arranged by electrocatalysts)

Nanoframe	Reaction system	Control of reduction kinetics	Edge thickness (edge length, selectivity)	Refs.
Five-fold-twinned PtCu NFs or PtCu ONFs	Glycine, PVP, NaI, H_2_PtCl_6_, CuCl_2_, ethanolamine aq., in an autoclave at 160 °C for 16 h or 200 °C for 2 h	Glycine	~ 5 nm (~ 60 nm, 80%); ~ 5 nm (~ 30 nm, 85%)	[205, 203]
PtCu RDNF/C (EM)	Pt(acac)_2_, CuCl_2_, glucose, OAm/OAc (the volume ratio is 4/1) at 180 °C for 3 h	High concentration of CuCl_2_	~ 2.3 nm (~ 17 nm, –)	[204]
PtCu RDNFs	Pt(acac)_2_, CuCl_2_, DGA, CTAC, OAm, in an autoclave at 180 °C for 10 h	DGA and CTAC	– (17.12 nm, –)	[206]
PtCu CONFs, UONFs, RDNFs, or CNFs, PtCuNi ONFs	H_2_PtCl_6_ (or Pt(acac)_2_ for CONFs), Cu(acac)_2_ (CONFs or ONFs, Cu(NO_3_)_2_ for UONFs or RDNFs, CuCl_2_ for CNFs), Ni(acac)_2_, CTAB (CTAC for RDNFs), OAm, in an autoclave at 170 °C for 24 h with moderate magnetic stirring	CTAB or CTAC, metal precursor (O_2_/Br^−^ or O_2_/Cl^−^ oxidative etching)	– (20.7 nm, 100%, CONFs); ~ 2 nm (14.1 nm, 100%, UONFs)	[208, 334]
PtCuMn NFs	Threonine, PVP, NaI, H_2_PtCl_6_, MnCl_2_, Cu(NO_3_)_2_, ethanolamine aq., in an autoclave at 160 °C for 16 h	Threonine, NaI, and ethanolamine	1.8 nm or 18.3 nm (–)	[207]

EM: ex situ mixing; ONFs: octahedral nanoframes; glycine: NH_2_CH_2_COOH; RDNFs: rhombic dodecahedral nanoframes; DGA: diglycolamine (NH_2_CH_2_CH_2_OCH_2_CH_2_OH); CONFs: concave octopod nanoframes; UONFs: ultrathin octopod nanoframes; CNFs: cubic nanoframes; Threonine: CH_3_CH(OH)CH(NH_2_)COOH

Typically, the galvanic replacement reaction and oxidative etching occurs simultaneously during the formation of NCgs or NFs due to the existence of O_2_ when synthesis is conducted in air [191, 208]. As shown in Tables 10, 11 and 13, the reaction systems for preparing NCgs or NFs are commonly not protected by an inert gas such as N_2_ or Ar. The presence of coordination ligands such as halide ions or N-containing organic molecules introduced by metal precursors, solvents, or structure-capping agents, combined with O_2_ often results in the etching of non-Pt atoms.

Considering the formation of Pt-based alloys, NCgs and NFs together, a conclusion can be drawn. Even though Pt ions commonly have a higher reduction potential than non-Pt ions, Pt and non-Pt ions can be reduced simultaneously, and even non-Pt ions can be reduced first by selecting suitable precursors, ligands, or reductants to vary their reduction kinetics or reduction potentials. The former forms Pt-based alloys, and the latter forms Pt-based core@shell structures, NCgs or NFs.

##### Chemical Etching

Pt-based polyhedrons with Pt-rich frameworks filled with Pt-poor alloys or non-Pt metals are prepared first, and then the interior and side-face non-Pt metal atoms are etched away (Table 14). Phase-segregated Pt-based polyhedrons are obtained by selectively depositing Pt atoms at the vertexes and edges of Pt-poor or non-Pt metal polyhedrons or selectively migrating Pt atoms to the vertexes and edges of Pt-based polyhedrons [202, 209–212]. For example, Pt atoms selectively deposit at the edges of Pd-rich nanocubes in the presence of Cu^2+^ species and I^−^, and at a relatively low reaction temperature [211]. It is believed that the surface energy is modified due to the deposition of Cu^2+^ on the side faces of Pd nanocubes so that the deposition sites of Pt^4+^ are altered, i.e., the deposition of Pt^4+^ preferentially occurs at edges. The relatively low reaction temperature and I^−^ facilitate the preferential deposition of Pt atoms at edges by affecting the Pt deposition process and suppressing the atomic diffusion of Pt.

**Table 14:** Summary of the methods for preparing nanoframes (NFs) by etching phase-segregated Pt-based polyhedrons (arranged by electrocatalysts)

Nanoframe	Precursor	Synthesis strategies of precursor	Etching methods	Wall thickness (size, loading)	Refs.
Pt OSNF/C (EM)	Octahedral PtNi	Pt(acac)_2_, Ni(acac)_2_, stearic acid, and octadecylamine, kept at 170 °C for 5 h under 1 atm (1 atm = 101 325 Pa) of CO gas	Acetic acid, 100 °C for 1 h in air	–	[209]
Pt_82_Co_18_ RDNF/C (EM)	Pt_23_Co_77_ RD	H_2_PtCl_6_, (CH_3_COO)_2_Co, OAm/OAc (4/2) at 240 °C for 8 min in N_2_	2 M HNO_3_, stirred at 60 °C for 1 h in air	– (~ 23 nm, 8–10 wt_Pt_%)	[335]
Pt-Co ND-NF/C (EM)	Pt-Co ND-SD	Co(acac)_2_, H_2_PtCl_6_, CTAB, OAm/OAc/H_2_O (10/2.5/1), in an autoclave and stirred at 180 °C for 24 h,	Br^−^ and O_2_ (H_2_O) (in situ etching)	–	[336]
Pt_3_Co NFs	PtCo_3_	Addition of H_2_PtCl_6_, (CH_3_COO)_2_Co, H_2_O, and OAm (60 °C, in N_2_) into OAm/OAc (the volume ratio is 5/2, 180 °C, in Ar), kept at 180 °C for 45 min, then at 240 °C for 2 min in Ar	Chloroform, hexadecane, OAm at 130 °C for 6 h in air	–	[337]
PtCu_3_ RDNF/C (EM)	PtCu_5_ RD	Pt(acac)_2_, Cu(HCOO)_2_, CTAC, OAm, kept at 160 °C for 5 h	FeCl_3_ and OAm at 100 °C for 1 h	– (~ 20 nm, –)	[338]
PtCuCo RDNF/C (EM)	Co-PtCu RD	Pt(acac)_2_, Cu(OAc)_2_, CoCl_2_, AA, OAm, heated at 80 °C for 5 min and then at 250 °C for 30 min in Ar	Acetic acid/ethanol (the volume ratio is 5/10)	– (~ 22.2 nm, 12.2 wt_Pt_%)	[339]
Pt_3_Ni RDNF/C (EM)	PtNi RD	Addition of H_2_PtCl_6_, Ni(NO_3_)_2_ and OAm (0.8 mL) into OAm (9.2 mL) at 160 °C in Ar, after being kept under vacuum for 2.5 min and then kept at 265 °C for 5 min in Ar	Toluene, acetic acid and OAm at 90 °C for 2 h	– (–, 15–18 wt_Pt_%)	[210]
Pt_3_Ni RDNF/C (EM)	PtNi_3_ RD	H_2_PtCl_6_, Ni(NO_3_)_2_, OAm, heated at 60 °C for 2–3 min and then at 270 °C for 3 min in Ar	Chloroform, hexadecane, OAm at 120 °C for 12 h in air	~ 2 nm (~ 20.1 nm, 20 wt%)	[21]
Pt_3_Ni THHNF/C (EM)	PtNi_4_ THH	PtCl_4_, NiCl_2_, OAm, ODE, heated at 180 °C for 10 min and then at 290 °C for 5 min in Ar	CO at 170 °C for 45 min	– (~ 22 nm, –)	[202]
PtNi D@F/C (EM)	PtNi nanostructure	Pt(acac)_2_, Ni(acac)_2_, CTAC, OAm, heated at 50 °C for 7 min and then at 270 °C for 30 min	Toluene, ethanol, and HCl, 60 °C for 1 h	2–3 nm (72.18 nm, 5 wt_Pt_%)	[214]
PtPdCu NFs	PtPdCu CNCs	PdCl_2_, KI, PVP, H_2_PtCl_6_, CuCl_2_, DMF, in an autoclave at 160 °C for 6 h	KBr, PVP, FeCl_3_ & HCl aq., 100 °C for 4 h	–	[211]

EM: ex situ mixing; OSNFs: octahedral skeletal nanoframes; RDNFs: rhombic dodecahedral nanoframes; ND-NF: nanodendrite in nanoframe; ND-SD: nanodendrite in solid rhombohedral dodecahedron; THHNFs: tetrahexahedral nanoframes; D@F: dendrite@frame; CNCs: concave nanocubes

The phase segregation of components can be characterized by XRD and TEM [202, 212]. In the XRD patterns, asymmetric or split diffraction peaks appear for phase-segregated Pt-based electrocatalysts and then turn into a single set of symmetric peaks after chemical etching. Regarding the case of Pt-rich frameworks filled with non-Pt metal, the contrast of vertexes and edges is brighter in TEM dark field images and darker in TEM bright field images.

Therefore, why will Pt selectively deposit at the edges instead of undergoing epitaxial growth? Two reasons might account for this. One is that side faces are occupied by some species so that Pt has no choice but to deposit at edges. The other is that to minimize the overall Gibbs energy or relieve internal strain arising from different atomic sizes, elements with a lower surface energy or larger diameter are more likely to segregate to the surface [212]. Taking {110}-closed rhombic dodecahedral PtNi with a Pt-rich framework as an example, Ni preferentially segregates to faces due to lower surface energies (Pt{110} and Ni{110} are 2.819 and 2.368 J m^−2^, respectively). However, the interior atoms and larger Pt atoms (2.13 Å (Pt) and 1.97 Å (Ni) [213]) tend to migrate to the vertex and edge sites of the dodecahedron to release internal strain [212]. Different from the synthesis of NCs, to prepare NFs, the non-Pt metal probably needs to have a much smaller atomic size than Pt, e.g., Ni, Co (2.00 Å), or Cu (1.96 Å) instead of Pd (2.10 Å) [213], thereby increasing the degree of lattice mismatch, suppressing the epitaxial growth of Pt on non-Pt or Pt-poor polyhedrons, and likely improving the migration of Pt atoms to the edges.

In addition, to facilitate atom migration, a relatively high reaction temperature or surface binding moieties are necessary. The reaction temperature can influence the degree of phase segregation by modulating the mobility of interior Pt atoms [214]. If the reaction temperature is not sufficiently high to overcome the energy barrier, the interior Pt atoms cannot migrate out to the more favorable surface sites. When CO gas is introduced, the migration energy barrier is decreased due to strong metal–CO bonds, thus leading to the successful outward diffusion of the embedded Pt atoms [209].

### Synthesis of 1D Pt-Based Nanostructures

**Fig. 11:**
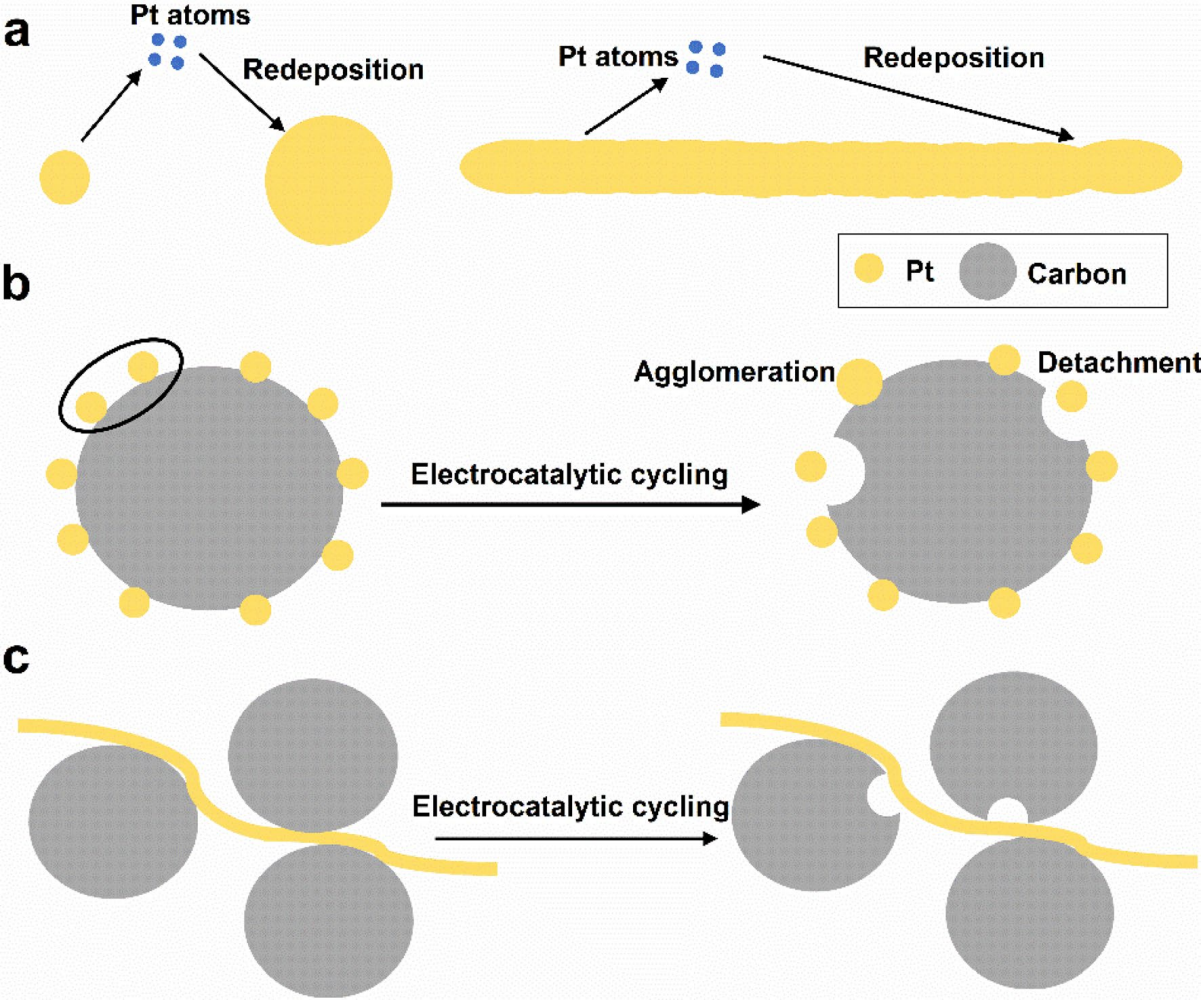
Schematic illustration of the Ostwald ripening process **a** of 0D Pt nanoparticles and 1D Pt NWs. Modified with permission from Ref. [40]. Copyright © 2016, the American Association for the Advancement of Science. The detachment or agglomeration of **b** 0D Pt nanoparticles or **c** 1D Pt NWs supported on carbon during electrocatalytic cycling

Zero-dimensional (0D) Pt-based nanoparticles supported on carbon always degrade through dissolution, Ostwald ripening, agglomeration, or detachment from carbon during electrochemical cycling, leading to a gradual decrease in the ECSA and electrocatalytic activity. To enhance the durability of Pt-based electrocatalysts in fuel cells, 1D Pt-based electrocatalysts, such as Pt-based nanowires (NWs), nanorods (NRs), nanofibers, nanotubes (NTbs), and nanowire networks (NNWs), have drawn extensive attention owing to the multiple advantages associated with their unique 1D morphology. First, the inherently anisotropic structure suppresses serious loss of the ECSA during the Oswald ripening process. Regarding 0D nanoparticles, the dissolved Pt atoms from small nanoparticles tend to redeposit on nanoparticles that are large, which are energetically favorable sites because of their smaller curvature. This gradually increases the size of the Pt nanoparticles and decreases the surface area on a per unit mass basis. However, dissolved Pt atoms from the 1D nanostructure will preferentially redeposit in the region with a negative curvature to reduce the total energy, helping to prevent the breakup of the 1D structure and thus stabilize it (Fig. 11a) [40]. Second, compared with a single point contact of Pt-based nanoparticles with carbon, multiple anchoring points of 1D Pt-based nanostructures with carbon strengthen the interaction between the metal and carbon support, not only improving electron transport but also suppressing the agglomeration and detachment of Pt-based electrocatalysts (Fig. 11b and c) [175, 215, 216].

In general, the synthesis methods for 1D Pt-based nanostructures include seed-mediated growth, the oriented attachment of small nanocrystals, and 1D hard templates (Tables 15, 16, and 17), which are summarized below.

**Fig. 12:**
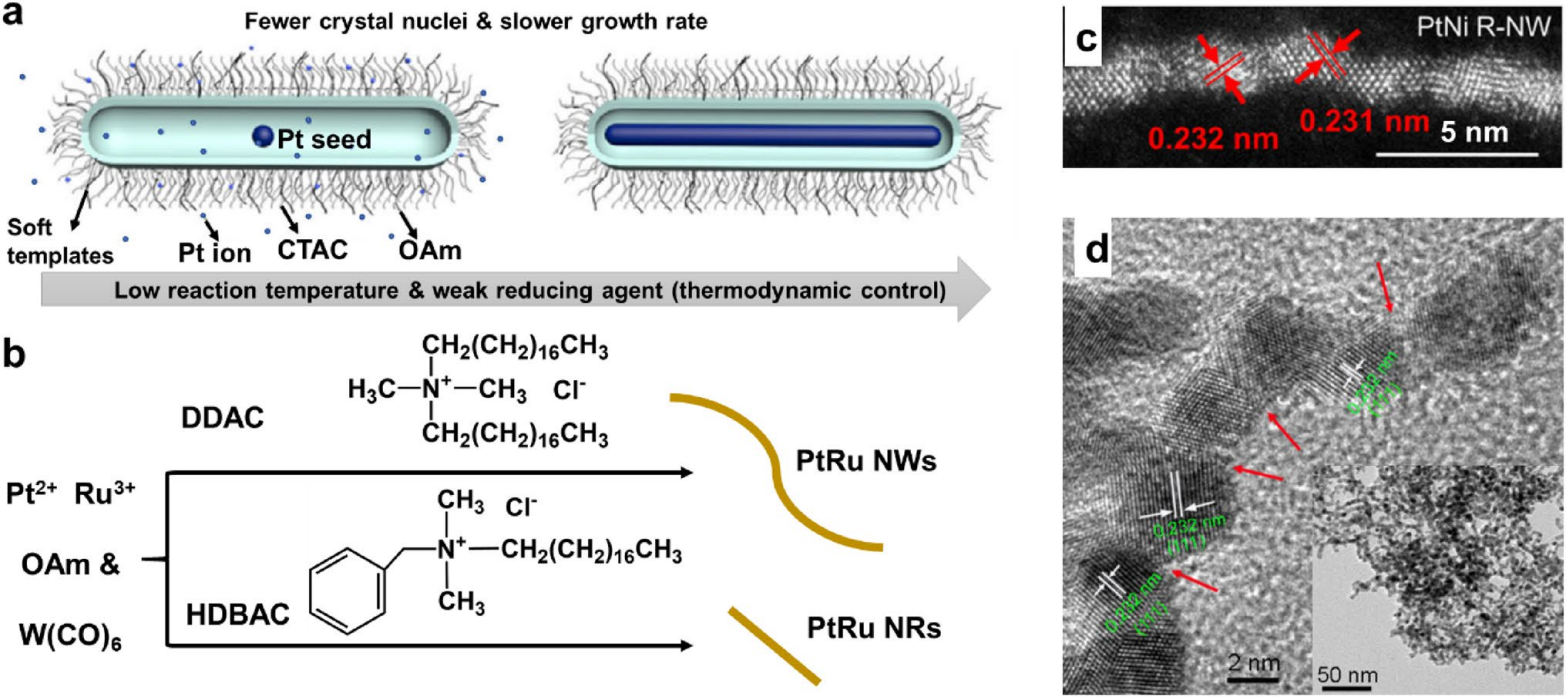
**a** Schematic illustration of Pt seeds growing into NWs inside the soft template formed with CTAB and OAm. Adapted with permission from Ref. [256]. Copyright © 2019, Springer. **b** Schematic illustration of PtRu NWs and PtRu NRs by using DDAC with double alkyl chains and HDBAC with only one alkyl chain as structure-directing agents, respectively [218]. **c** HAADF-STEM image of PtNi NWs prepared by the oriented attachment formation mechanism. Adapted with permission from from Ref. [256]. Copyright © 2019, Springer. **d** HRTEM image of individual AgPt NWs in NNWs, the inset: the LRTEM image of AgPt NNWs. Adapted with permission from Ref. [228]. Copyright © 2018, Springer

1. *Seed-mediated growth*. Pt-based seeds can gradually grow into 1D nanostructures by confining the reduction of metal precursors in soft templates or under the control of structure-directing agents (such as HCOOH [39, 217]). Soft templates primarily refer to reverse micelles with 1D structures that form with N-containing surfactants (such as dimethyldioctadecylammonium chloride (DDAC), hexadecyldimethylbenzyl ammonium chloride (HDBAC) [218], CTAB [219, 220], didecyldimethylammonium bromide (DDAB) [175], CTAC, or octadecyltrimethylammonium chloride (STAC) [221]) in organic solvents (such as OAm [220] or chloroform [144]), which can guide the anisotropic growth of seeds into 1D nanostructures (Fig. 12a). The length and aspect ratio of 1D Pt-based nanostructures can be controlled by varying the concentration of metal precursors [144], the species and concentration of surfactants (e.g., surfactants with a different carbon number of their alkyl chain), solvents, and reaction temperature [218, 221]. For example, if HDBAC is used with only one alkyl chain, PtRu NRs are prepared, and the length of the NRs decreases with an increase in the amount of HDBAC due to the shortened micelles; in contrast, if DDAC is used with a double alkyl chain, longer and stable micelles might be formed, prolonging anisotropic growth into nanowire structures (Fig. 12b) [218]. The aspect ratio of PtSn NFs changes from 13.4 to 22.5 by simply tuning the species of surfactant (STAC or CTAC) and solvent (OAm or OAm/ODE) [221]. It is worth noting that 1D Pt-based nanostructures supported on carbon by a one-pot method are rarely synthesized by using soft templates, probably because the adsorption of surfactants on carbon will disturb the formation of 1D reverse micelles [30, 144].
  In addition, zigzag-like PtM (M = Ni, Zn, Cu, Fe, or Co) NWs have recently been reported due to their rough surface with abundant high-index facets or coordinatively unsaturated sites, which might have higher electrocatalytic activity [215, 222–226]. The formation of zigzag-like NWs generally relies on the initial formation of ultrathin Pt NWs with smooth surfaces due to the different reduction potentials of Pt and M, followed by the reduction of non-Pt metal onto Pt NWs. The reduced M atoms diffuse into the Pt lattice, forming an alloy phase, during which zigzag-like nanostructures are formed due to the different diffusion rates of Pt and M. The size of the humps can be controlled by adding different amounts of the M precursor.
2. *Oriented attachment of small nanocrystals*. 1D Pt-based electrocatalysts can be obtained by the continuously oriented attachment and coalescence of initially formed Pt-based nanoparticles. Such 1D nanostructures generally have a rough surface or a wavy structure and an even diameter due to the existence of a necking area between the Pt-based nanoparticles; thus, they always show a network-like structure composed of interwoven NWs (Fig. 12c and d) [227–230]. Oriented attachment may result from the confined function of soft templates or the different adsorption properties of structure-capping agents (such as H_2_, amine-terminated poly(*N*-isopropyl acrylamide (PNIPAM-NH_2_), PVP or Br^–^) on the different facets of Pt-based nanoparticles [98, 216, 228, 229, 231]. For example, PNIPAM-NH_2_ preferentially adsorbs on the {100} and {110} facets of Pt or PtAg nanoparticles, while a smaller amount of PNIPAM-NH_2_ adsorbs on the {111} facets, resulting in attachment preferably occurring on the {111} surfaces and the 1D nanostructures growing along the < 111 > orientation [228].
3. *Hard template*. Pt-based NTbs can be prepared by depositing Pt onto 1D templates and then removing the templates by chemical etching [232].

**Table 15:** Summary of the methods for preparing 1D Pt-based nanostructures by seed-mediated growth (arranged by the type of solvent)

Catalyst	Precursor	Solvent^a^; reductant	Capping agent	Reaction conditions	Diameter, length (aspect ratio)	Refs.
PtRu NWs	Pt(acac)_2_, Ru(acac)_3_	OAm; W(CO)_6_	DDAC (or HDBAC)	185 °C for 5 h in N_2_	1.8 nm, 130 nm (DDAC); 2 nm, 65, 45 or 30 nm (HDBAC)	[218]
Pt or PtRh NW/C (EM)	Pt(acac)_2_, Rh(acac)_3_	OAm; W(CO)_6_	CTAB	190 °C for 5 h	1.2 nm, 30 nm (PtRh)	[220]
Pt or PtRh NW/C (EM)	Pt(acac)_2_, Rh(acac)_3_	OAm; W(CO)_6_	DDAB	Addition of W(CO)_6_ at 180 °C and then kept for 2 h	1.8 nm, 38.3 nm (Pt); 1.3 nm, 40.4 nm (PtRh)	[175]
Pt, PtNi, or PtNiRh NWs	Pt(acac)_2_, Ni(acac)_2_, Rh(acac)_3_	OAm; Mo(CO)_6_	CTAB	Addition of Mo(CO)_6_ at 170 °C and then kept for 2 h	2.1 nm, 50 nm (Pt); 1.2 nm, 60 nm (PtNi); 1 nm, 80 nm (PtNiRh)	[219]
Pt, or PtCo/C (EM)	Pt(acac)_2_, Co(acac)_2_	OAm; Cr(CO)_6_	OAm & Cr(CO)_6_	In an autoclave at 180 °C for 5 h	2 nm, 30 nm	[340]
PtM (M = Ni, Zn, Cu, Fe, Co) zigzag-like NWs	Pt(acac)_2_, M(acac)_*x*_	OAm; glucose (or OAm)	CTAC (or CTAB)	160, 180 or 200 °C for at least 1 h	–, 4–24 nm	[215, 222, 224–226]
PtSn NF/C (EM)	Pt(acac)_2_, Sn(Ac)_2_	OAm (NFs-L or S), ODE/OAm (1/4, NFs-M); Mo(CO)_6_	STAC (NFs-L or M), CTAC (NFs-S)	180 °C for 5 h	1.7 nm, 38.2 nm (22.5, NFs-L);–(17.5, NFs-M);–(13.4, NFs-S)	[221]
Pt, PtNi, PtCo or PtNiCo NWs	Pt(acac)_2_, Ni(acac)_2_, Co(acac)_2_	OAm/DMF (8/10); H_2_/CO (1:1)	Synergistic effect of CTAB and OAm	120 °C for 7 h	0.6–1.8 nm, –	[256]
Pt or Pt_3_Ni NWs	H_2_PtCl_6_, Ni(acac)_2_	EG/DMF (1/1, KOH); –	− NH_2_ (DMF)	In an autoclave at 170 °C for 8 h	1 nm, $\geqslant$ 100 nm (Pt_3_Ni); 1 nm,–(Pt)	[256]
Pt_9_Co_1_ NW/C (EM)	H_2_PtCl_6_, CoCl_2_	HCCl_3_/H_2_O (1/18); NaBH_4_	CTAB	1 000 rpm for 20 min	(2.2 ± 0.2) nm, –	[341]
Pt NW/C (OP)	H_2_PtCl_6_	H_2_O; HCOOH	HCOOH	72 h	4 nm, 10–30 nm	[39, 217]

^a^The ratio of mixed solvent is in volume ratio

EM: ex situ mixing; OP: one pot; DDAC: dimethyldioctadecylammonium chloride; HDBAC: hexadecyldimethylbenzyl ammonium chloride; DDAB: didecyldimethylammonium bromide; STAC: octadecyltrimethylammonium chloride (CH_3_(CH_2_)_17_ N(Cl)(CH_3_)_3_); NFs-L, M, or S: nanofibers with a large, medium, or small aspect ratio

**Table 16:** Summary of the methods for preparing 1D Pt-based nanostructures by the oriented attachment formation mechanism (arranged by the type of solvent)

Catalyst	Precursor	Solvent^a^; reductant	Capping agent	Reaction conditions	Diameter, length (aspect ratio)	Refs.
Pt, PtNi, PtCo or PtNiCo NWs	Pt(acac)_2_, Ni(acac)_2_, Co(acac)_2_	OAm; W(CO)_6_	Synergistic effect of CTAB and OAm	Addition of W(CO)_6_ at 130 °C, then kept at 240 °C for 45 min	0.6–2 nm, –	[256]
Pt NW/C (EM)	Pt(acac)_2_	OAm; OAm	Ni^2+^ (Ni(acac)_2_), CTAC, carbonyl (Mo(CO)_6_)	160 °C for 2 h	0.8 nm,–(controlling the length by changing the amount of Mo(CO)_6_)	[342]
PtNi or PtNiCo NW/C (EM)	Pt(acac)_2_, Ni(acac)_2_, Co(acac)_2_	OAm; glucose	CTAC, carbonyl (Mo(CO)_6_)	160 °C for 2 h	0.8 nm, 120 nm (PtNi); 0.8 nm,–(PtNiCo)	[342]
PtPdCu NNWs	H_2_PtCl_6_, PdCl_2_, CuCl_2_	DMF; DMF	Br^−^ (KBr), PVP	In an autoclave at 200 °C for 4 h	5–8 nm, –	[229]
Pt NWs/SG or Pt NW/S-CNT (OP)	H_2_PtCl_6_	EG/DMF (2/3 or 1/1, KOH); –	–	In an autoclave at 170 °C for 8 h	3–28 nm, 1 µm	[98, 343]
PtSn NNWs	K_2_PtCl_4_, SnC_2_O_4_	EG; phenol	PVP	160 °C for 5 h and then at 180 °C for 2 h	–	[344]
PtCu NNW/C (EM)	K_2_PtCl_4_, CuBr_2_	H_2_O/EG (1/1); EG	Na_2_H_2_P_2_O_7_	110 °C for 3 h	2.4 nm, –	[230]
PtPd NNWs	H_2_PtCl_6_, Na_2_PdCl_4_	H_2_O; NaBH_4_	Br^–^ (KBr), PVP	30 min	5 nm, –	[227]
PtCu NNW/RGO (OP) or PtAg NNWs	H_2_PtCl_6_, CuCl_2_, AgNO_3_	H_2_O; HCHO	PNIPAM-NH_2_	In an autoclave at 140 °C for 6 h	2.4 nm, – (PtCu); 3.9 nm, – (PtAg)	[216, 228]
Pt or PtNi NW/C (OP)	K_2_PtCl_4_, NiCl_2_	–	H_2_	Impregnation and reduced by H_2_ at 250 °C for 30 min	3 nm, –	[231]

^a^The ratio of mixed solvent is in volume ratio

EM: ex situ mixing; OP: one pot; PNIPAM-NH_2_: amine-terminated poly(*N*-isopropyl acrylamide); SG: S-doped graphene

**Table 17:** Comparison of the three synthesis methods for 1D Pt-based nanostructures

Method	Advantages	Disadvantages
Seed-mediated growth	No necking area, more stable Zigzag-like NWs, abundant high-index facets, high activity	–
Oriented attachment of small nanocrystals	–	Fragile during ultrasonic dispersion due to the presence of necking between the two attached particles Easily forms a network, which is adverse to loading onto a support
Hard template	Forming a metal tube simultaneously exposes the internal and external surfaces	Removing the hard template commonly needs harsh conditions

### Synthesis of 2D Pt-Based Nanostructures

In addition to 1D Pt-based electrocatalysts, 2D Pt-based nanosheets (NSs) or nanoplates (NPs) have also been increasingly studied among various anisotropic structures due to their unique physicochemical properties induced by their large lateral-to-thickness aspect ratio [20, 233, 234]. Ultrathin 2D Pt-based nanostructures have not only a large surface area but also the largest contact area with carbon in comparison with 0D nanoparticles and 1D nanostructures, resulting in enhanced electrocatalytic activity and durability [20, 235, 236]. However, unlike intrinsically layered materials, such as graphene, hexagonal boron nitride, or MoS_2_, which have covalent bonds in a layer and weak van der Waals forces between layers, the face-centered cubic (fcc) structure of metallic Pt is a highly symmetric crystal lattice with strong nondirectional metallic bonding in 3D [237–239]. 2D-shaped Pt is thermodynamically unfavorable during growth since a symmetry-breaking event takes place for anisotropic growth. To prepare 2D Pt-based nanomaterials, many strategies have been investigated, which mainly include two categories: top-down approaches and bottom-up approaches. Regarding the top-down approach, 2D Pt can be synthesized only by mechanical compression (e.g., heat-pressing processes [240]) and not through exfoliation, such as intrinsically layered materials. Regarding the bottom-up approach, 2D Pt-based nanostructures can be prepared by suppressing growth in the thickness direction and aiding lateral growth, such as using soft or hard templates (2D structural micelles, reverse micelles, or 2D substrates) or capping agents to regulate growth, as well as forming hexagonal close-packed (hcp) Pt-based alloys (Table 18). The details are presented below.

1. *2D soft or hard templates*. 2D Pt-based nanostructures can be prepared by confining the nucleation and growth of Pt-based nanocrystals in 2D micelles or reverse micelles as well as in the interlayer spaces of layered materials [234, 241, 242], by confining the growth of Pt-based nanocrystals on the surface of 2D materials with functional groups on the surface [243], or by the epitaxial growth of Pt on 2D metal [244, 245]. A gel-like material prepared with PVP and tris(hydroxymethyl)aminomethane in HCHO is used as a template to confine the nucleation and growth of PtCu or PtAg, forming Pt-based NSs [153, 246]. Surfactants, such as CTAB and sodium perfluorooctanoate (FC7), can assemble into bicellar-like micelles in water, which are used to control the growth of Pt, thereby forming Pt nanodisks or nanowheels [241]. When docosyltrimethylammonium chloride (C_22_TAC) is used as a surfactant, the hydrophilic quaternary ammonium head binds with the metal precursors (PdCl_4_^2–^ or PtCl_6_^2–^) by electrostatic interactions, and the long-chain hydrophobic C_22_ tail drives self-assembly into a stabilized lamellar mesophase, causing the in-the-plane reduction of metal complexes into ultrathin 2D Pt-based nanostructures in situ [236]. Due to the existence of abundant oxygen-containing functional groups, Pt nanoparticles assemble into a sheet-like aggregate due to the immobilization function of GO and then form Pt NSs by Ostwald ripening [184, 243].
2. *Capping agent-mediated growth*. As mentioned in Sect. 4.2, capping agents can specifically adsorb on different facets of different metals, which is used to tune the shape. CO can suppress growth in the thickness direction by its strong absorption on basal {111} planes. Therefore, Pt-based NSs are successfully synthesized by the introduction of a second metal (such as Cu or Pd) to alter the preferential adsorption of CO on {100} facets to {111} facets [237, 245, 247, 248].
3. *Hexagonal close-packed (hcp) Pt-based alloys*. In addition to the function of templates or capping agents, the final shape of the crystal also relies on its own crystal structure. Pure Pt tends to form polyhedrons closed by {111} or {100} facets due to its fcc structure. Different from the fcc structure of pure Pt or conventional Pt alloys, PtBi has an hcp-structured intermetallic phase. By utilizing this uniaxial crystal structural character of PtBi, PtBi NPs can be successfully synthesized by choosing Br^–^ as a capping agent, which preferably adsorbs on the (101) plane of PtBi [235, 249].

**Table 18:** Summary of the methods for preparing 2D Pt-based nanostructures by the bottom-up approach (arranged by the type of solvent)

Catalyst	Precursor	Solvent^a^; reductant; stabilizer	Reaction conditions	Factors influencing shape	Thickness (lateral size)	Refs.
PtPdM (M = Ni, Co or Fe) NS/C (EM)	Pt(acac)_2_, Pd(acac)_2_, M(acac)_*x*_	OAm; –	160 °C for 1 h	Pd, CO (Mo(CO)_6_)	1.4 nm (–)	[345]
PtBi NP/C (EM)	Pt(acac)_2_, Bi(NE)_3_	OAm; –	90 °C for 30 min, then at 200 °C for 5 min in N_2_	Bi and Br^–^ (NH_4_Br)	6 nm (40 nm)	[235]
PtRu or PtRuM (M = Fe, Co, Ni) NS/C (EM)	Pt(acac)_2_, Ru(acac)_3_, M(acac)_*x*_	OAm; –; CTAB	Addition of Mo(CO)_6_ at 200 °C and then kept for 2 h	CO (Mo(CO)_6_)	2 nm (6.2 nm)	[346]
PtPb@Pt NP/C (EM)	Pt(acac)_2_, Pb(acac)_2_	OAm/ODE (1/1); AA; PVP	160 °C for 5 h	Synergy of the precursor, reductant, and solvent	4.5 nm (~ 16 nm)	[20]
PtBi@Pt NP/C (EM)	Pt(acac)_2_, Bi(Ac)_3_	ODE/OAm (1/1); AA; –	160 °C for 5 h	Br^–^ (NH_4_Br), solvent, and the ratio of Pt/Bi	4.6 nm (–)	[347]
PdPtCu or PdPt NS/C (EM)	Pd(acac)_2_, Pt(acac)_2_, Cu(acac)_2_	DMF; CA; PVP	80 °C and 320 rpm for 3 h, and then at 150 °C for 3 h	Initially, formed Pd NSs in the presence of Br^–^ (KBr) and CO (Mo(CO)_6_)	~ 2.2 nm (7.3 nm)	[245]
PtPd NSs	H_2_PtCl_6_, Na_2_PdCl_4_	DMF/EG/DETA (6/4/5); –	In an autoclave at 200 °C for 2 h	DETA and DMA (decomposition of DMF induced by KOH)	2 nm (1–2 µm)	[348]
Pd@PtCu NP/C (EM)	Pd NPs, Pt(acac)_2_, Cu(acac)_3_	Benzyl alcohol; –; PVP	In an autoclave at 200 °C for 12 h	Pd NPs	3.5–4 nm (10 nm)	[244]
PtTe NS/C (EM)	Pt(acac)_2_, Te(OH)_6_	DMAC; CO (1 bar)/HCOOH; –	160 °C for 2 h	CO/HCOOH	0.9 nm (–)	[248]
PtCu NSs	Pt(acac)_2_, Cu(acac)_2_	Formamide; –; PVP	CO (1 bar), 130 °C for 3 h	Cu, CO, I^–^ (KI)	~ 1.6 nm (13 nm)	[247]
PtCu or PtAg NSs	Pt(acac)_2_, Cu(acac)_2_, AgNO_3_	Formamide; HCHO (or AA); –	In an autoclave at 130 °C for 3 h	Gel-like material (PVP, Tris, HCHO), I^–^ (KI), CO (HCHO)	0.17–1.32 nm (10–50 nm)	[153, 246]
PtPd or Pt NSs/RGO (OP)	K_2_PtCl_4_, Na_2_PdCl_4_	KNO_3_-LiNO_3_ molten salt; H_2_; –	200 °C for 1 h	GO	10 nm (20–75 nm, PtPd); 9.8 nm (50–100 nm, Pt)	[184, 243]
PdPtAg NSs	K_2_PdCl_4_, K_2_PtCl_4_, AgNO_3_	CO saturated H_2_O; AA; CTAC	95 °C for 2 h	Pd, CO	3 nm (~ 69 nm)	[237]
PdPtCu NSs	H_2_PdCl_4_, H_2_PtCl_6_, Cu(NO_3_)_2_	H_2_O; AA; –	Room temperature for 30 min	C_22_TAC	~ 3.5 nm (~ 45 nm)	[236]
2D Pt NDs	H_2_PtCl_6_	H_2_O; AA; –	50 °C for 12 h	C_22_N-COOH(Br^–^)	2.5 nm (150 nm)	[349]

^a^The ratio of mixed solvent is in volume ratio

EM: ex situ mixing; OP: one pot; Bi(NE)_3_: bismuth(III) neodecanoate; Bi(Ac)_3_: bismuth(III) acetate; CA: citric acid; DETA: diethylenetriamine; DMA: dimethylamine; DMAC: *N*,*N*-dimethylacetamide; Tris: tris(hydroxymethyl)aminomethane; NDs: nanodendrites; C_22_TAC: docosyltrimethylammonium chloride; C_22_N–COOH(Br^–^): C_22_H_45_–N^+^(CH_3_)_2_CH_2_COOH (Br^–^)

## Postsynthesis Treatments of Pt-Based Electrocatalysts

Many reaction systems have been developed to synthesize Pt-based electrocatalysts, particularly wet chemical methods. To tailor the final size, size distribution or shape, the reaction conditions as well as the kind and concentration of organic or inorganic metal precursors, reductants, structure-directing agents (polymers, surfactants, or halide ions), stabilizers, or solvents (water or organic solvents) should be carefully singled out. In a specific reaction solution, there may be more than one reagent (hereafter collectively referred to as “ligands”) adsorbing on or even binding to the surface of Pt-based electrocatalysts to control the shape and size, protecting Pt-based nanoparticles from aggregation, or only adsorbing on their surface without providing any benefit. These ligands still exist on the surface of Pt-based electrocatalysts after the reaction ends [250]. Although residual ligands can be utilized to improve catalytic performance in some cases [251, 252], it is necessary to remove them before applying catalysts to electrochemical reactions, since these ligands physically (e.g., cover the active sites or block the contact of reactants with Pt-based electrocatalysts) or chemically (e.g., detrimentally react with reactants or intermediates) hinder the electrocatalytic reaction [252, 253]. Hence, postsynthesis treatments are necessary and important and highly influential on the activity and durability of the final Pt-based electrocatalysts. After decontamination, more active sites are exposed, boosting the activity of Pt-based electrocatalysts [253]. Notably, an increase in particle size or size distribution, nanoparticle aggregation, altering of shape or composition, and atomic reconstruction may occur during postsynthesis treatment. Furthermore, atomic reconstruction of the surface during postsynthesis treatment may produce a Pt or Pt-rich shell outside Pt-based alloys, enhancing the durability of the electrocatalyst [254].

In general, synthesized Pt-based electrocatalysts are first purified with copious amounts of solvent. Frequently used solvents include water, ethanol, acetone, toluene, isopropanol, hexane, and cyclohexane or a mixture (at a certain volume ratio) of two of these [186, 226, 237, 255, 256]. Because different solvents or mixed solvents have different properties leading to different solubilities of ligands, a suitable solvent should be chosen to remove specific ligands [257]. However, only weakly attached ligands can dissolve into solvents from the surface of Pt-based electrocatalysts. Some strongly adsorbed organic ligands cannot be removed merely by washing with solvent [258].

Before developing a method to thoroughly clean Pt-based electrocatalysts, we should determine ways to characterize whether ligands remain on the electrocatalyst surface. Commonly used characterization techniques include IR spectroscopy, Raman spectroscopy, TGA, XPS, CV, and linear sweep voltammetry (LSV) [250, 258]. If organic ligands remain on the surface of Pt-based electrocatalysts, there will be some corresponding characterized peaks in the IR or Raman spectra, or weight loss peaks referring to the decomposition of residual ligands in the TGA curve. In addition, CV and LSV, or the ECSA and electrocatalytic activity, can be used as probes to detect whether the surface is completely exposed [30, 259]. For example, regarding Pt-based electrocatalysts with a clean surface, hydrogen adsorption and desorption peaks should appear in the first CV cycle and remain unchanged during the following CV cycles. Otherwise, the characterized adsorption/desorption peaks on the Pt surface will not appear during the initial cycles but will then appear or increase with increased electrochemical cycling due to the gradual removal of surface impurities [30].

Pt-based electrocatalysts with strongly adsorbed organic ligands need to be further purified. Before further purification, Pt-based electrocatalysts without supports are commonly first supported on carbon by ex situ mixing in some studies to prevent nanoparticle aggregation to some degree during the purification process. Recently, extensive efforts have been made to clean Pt-based electrocatalysts without significantly changing their shape, size, and dispersion. Generally used methods include but are not limited to acid washing, thermal annealing, UV-ozone irradiation, and electrochemical cleaning (Table 19); these methods are described below.

1. *Acid washing*. An acid–base reaction can be used to remove amine-containing ligands such as OAm or PVP from electrocatalysts [252]. Carbon-supported or nonsupported Pt-based electrocatalysts with amine-containing ligands are treated in pure acetic acid or its aqueous solution at approximately 60–70 °C for at least 30 min [150, 175, 183, 219, 260, 261]. Notably, the surface and subsurface transition metal will be leached out during the acid washing process, forming a Pt or Pt-rich shell and improving the stability of Pt-based alloys in fuel cells [254].
2. *Thermal annealing*. The residual organic ligands can be degraded by annealing in air at 180–350 °C for at least 1 h [65, 259, 262–265] or evaporated by annealing in an inert atmosphere, e.g., annealing in an Ar atmosphere at 300 °C for 1 h to remove EG [165]. However, size increases and shape modifications frequently occur during thermal annealing. Surface metal atoms are usually oxidized by air at elevated temperatures. Therefore, subsequent heat treatment in a reducing atmosphere, such as a mixed gas of H_2_ and N_2_, is commonly needed to ensure a metallic surface [65, 263, 265].
3. *UV-ozone irradiation*. Cleaning with UV-ozone irradiation is a photosensitized oxidation process [266]. The electrocatalysts are irradiated by UV light (wavelengths of ~ 185 nm and ~ 254 nm) at room temperature for a given period of time in air [68, 267]. O_2_ molecules absorb the UV light at a wavelength of ~ 185 nm and then dissociate, producing atomic oxygen or ozone. Moreover, organic ligands are excited and/or dissociated after absorbing the UV light at a wavelength of ~ 254 nm. Then, the excited ligands are decomposed to simpler volatile molecules through the oxidation of atomic oxygen or ozone [266]. Compared with thermal annealing, size increases and shape modifications can be partially inhibited by the UV-ozone method due to its low operating temperatures. Nevertheless, as light travels in straight lines, shielded organic ligands may not be eliminated. In addition, similar to thermal annealing, the surface is partially oxidized by atomic oxygen or ozone, e.g., the amount of Pd^*δ*+^ increases from the initial 12% to 54% based on XPS after 4 h of irradiation [266]. Some byproducts produced by the decomposition of organic ligands, e.g., CO, a product of the decomposition of PVP during UV-ozone irradiation, still remain on the surface of electrocatalysts [266].
4. *Electrochemical cleaning* [250, 268]. During electrochemical cycling, the Pt surface is oxidized, forming Pt–O bonds at high potentials; then, the hydrogen evolution reaction occurs on the Pt surface and forms Pt–H bonds at low potentials. The robust Pt–O or Pt–H bond can displace the Pt–ligand coordination bond due to its higher binding energy (Pt − O: 82.3 kcal mol^–1^, Pt − H: 75.1 kcal mol^–1^, Pt − OAm: 23.1 kcal mol^–1^, Pt-dodecanethiol: 17.7 kcal mol^–1^ or Pt-triphenylphosphine: 38.1 kcal mol^–1^) [268]. In addition, the oxidative decomposition of organic ligands occurs at high potentials. These organic ligands are gradually removed with increased electrochemical cycling. In addition, due to its stronger interactions with Pt-based electrocatalysts, CO can replace the PVP adsorbed on Pt-based electrocatalysts at low potentials and then be stripped at high potentials [211], thereby resulting in a clean surface.
5. *Other methods*. For example, Pt_3_Ni NCs have been subjected to Ar plasma treatment to remove residual organic solvent atoms and surfactants [179]. When a Pt nanoparticle suspension solution is mixed with H_2_O_2_/H_2_SO_4_ aq. and subsequently centrifuged, PVP is removed from the Pt surface by the O_2_ bubbles produced from the decomposition of H_2_O_2_ on the Pt surface [269]. In addition, PVP can also be removed from PtPd NCs by NaBH_4_/tert-butylamine (TBA) treatment [253]. The hydride generated from the hydrolysis of NaBH_4_ displaces PVP, resulting in PVP desorption. The desorbed PVP dissolves in TBA, an organic solvent in which PVP is highly soluble.

**Table 19:** Comparison of four postsynthesis treatments

Method	Advantages	Disadvantages
Acid washing	Simple Forms a Pt-rich shell, increasing the durability	Only suitable for amine-containing ligands
Thermal annealing	Simple Suitable for all organic ligands	Increases particle size and the size distribution Metal oxidation
UV-ozone irradiation	–	Needs expensive instruments Hardly removes whole ligands Metal oxidation
Electrochemical cleaning	Suitable for all surface impurities	Ligands can be removed from the metal surface, but still exist in the fuel cell

It is worth noting that the interaction between the different ligands and different metal surfaces may vary, which is determined by the chemical nature of ligands, the nature of the metal and the atomic arrangement on the surface [270]. Developing a universal cleaning procedure might be difficult. Pt-based electrocatalysts synthesized in different reaction systems need to be cleaned by specific approaches based on their particular case. Moreover, the cleaning method should be simple, time-saving, inexpensive, and environmentally friendly in terms of scaled-up production. Given that it is difficult to avoid the influence of postsynthesis treatment on Pt-based electrocatalysts, synthesis methods employing easily removable ligands or surfactant-free synthesis methods for Pt-based electrocatalysts need to be expansively developed. In addition, water-based reaction systems should be given more attention instead of organic reaction systems because organic solvent molecules commonly demonstrate strong absorption on electrocatalyst surfaces.

## Future Directions and Prospects

After prolonged endeavors worldwide, PEMFCs are now being commercially applied. However, as the most practical catalysts for PEMFCs, Pt-based electrocatalysts impede the commercialization of PEMFCs due to their high cost and limited supply. Improving the activity and durability of Pt-based electrocatalysts and thus decreasing their loading has become one of the crucial targets to reduce the cost of PEMFCs and achieve their widespread application. To improve their activity and durability, significant progress has been achieved regarding the controlled synthesis of carbon-supported Pt-based electrocatalysts. However, most synthesis progress is limited to the laboratory level. Much more effort toward experimental research and development is still needed to commercialize carbon-supported Pt-based electrocatalysts with high activity and durability and thus PEMFCs.

### Studies on the Functionalization of Commercial Carbon Supports

After functionalization, the number of nucleation sites or anchoring sites on commercial carbon support surfaces increases significantly, improving the dispersion of Pt-based electrocatalysts on carbon, making size control easier, and strengthening metal–support interactions. Pt-based electrocatalysts supported on functionalized carbon commonly show higher activity and durability than those supported on pristine carbon. Some perspectives on the existing challenges and future research directions of the functionalization of commercial carbon supports are described here.

1. To improve the hydrophilicity of carbon and thus promote the dispersion of carbon in polar solvents (e.g., water), positively or negatively charged groups can be introduced onto the surface. Regardless of which charged groups are introduced, the hydrophilicity of carbon is enhanced, and the size or dispersion of Pt-based electrocatalysts can be optimized by selecting metal complexes with the opposite charge. However, given the electrostatic interaction between the groups on the carbon and the sulfonate groups in the ionomer, the presence of positively charged groups on the carbon surface can facilitate the uniform distribution of ionomers on carbon-supported Pt-based electrocatalysts when making catalyst layers. Furthermore, regarding the preparation of Pt/C (e.g., commercial Pt/C), the introduction of positively charged groups onto the carbon of Pt/C may be an effective way to make the ionomer distribute uniformly on Pt/C. It is worth noting that the process that introduces positively charged groups onto Pt/C should not influence the size, shape, and activity of Pt nanoparticles.
2. Regarding heteroatom doping, the amount of doping is still low by ex situ methods, commonly less than 10 at% and even less than 1 at% in some cases, limiting the effect of heteroatoms on improving the performance of Pt-based electrocatalysts to some extent. Therefore, how to increase the amount of doped heteroatoms still needs to be investigated.
3. Regarding the introduction of metal oxides, it should be noted that the size of metal oxide nanoparticles on carbon should be sufficiently small to maximize the potential advantages of carbon–metal oxide composite supports and have no influence on electron transport between Pt-based electrocatalysts and carbon supports; this is still a challenge. Therefore, the development of new methods to decrease the size of metal oxide nanoparticles on carbon supports is still necessary. In addition to the introduction of metal oxides, the introduction of other noncarbon materials, e.g., nitrides and carbides, may be an effective way to functionalize carbon. The formation of porous ultrathin noncarbon material–metal oxide layers outside of carbon materials instead of nanoparticles probably maximizes the potential advantages of composite supports.

### Production of Pt-Based Electrocatalysts

Although a large number of nonspherical Pt-based electrocatalysts with high performance and durability have been reported in the literature, until now, commercial electrocatalysts are still spherical carbon-supported Pt-based electrocatalysts. More efforts need to be devoted to the mass production of well-defined nonspherical carbon-supported Pt-based electrocatalysts. Here, some probable reasons influencing their mass production capability are presented below, which is expected to help accelerate this process.

1. If the production scale is only made by scaling up the reaction system used in the laboratory, the prepared nonspherical Pt-based electrocatalysts commonly lose the control of their size and shape, thus showing worse performance and durability than those prepared in a small batch. Compared with spherical nanoparticles, the reaction systems for preparing nonspherical Pt-based electrocatalysts are generally more complex, which heightens the difficulty of keeping everything in the scaled-up reaction system uniform and consistent with the laboratory-scale system. Various structure-capping agents are necessary to control the morphology, which improves the difficulty of postsynthesis treatment. Furthermore, the mechanisms by which experimental parameters affect the size and shape of nonspherical Pt-based electrocatalysts are still not well understood and indistinct; thus, it is impossible to effectively control the size and shape when scaling up for mass production. Hence, it is necessary to scale up the production of nonspherical Pt-based electrocatalysts to develop simple reaction systems and understand the influencing mechanisms of every experimental parameter on the quality of the final synthesized electrocatalysts.
2. Although the size and shape can be relatively easily controlled for the ex situ mixing method, the one-pot synthesis method is more suitable for cost-effective mass production. In comparison with spherical carbon-supported Pt-based electrocatalysts, it is harder to prepare well-defined nonspherical carbon-supported Pt-based electrocatalysts by a one-pot method since the addition of carbon will disturb the control of the shape. Therefore, for mass production, significantly more efforts should be made to synthesize nonspherical carbon-supported Pt-based electrocatalysts by a one-pot synthesis method instead of an ex situ mixing method.
3. It is worth mentioning that most reported nonspherical Pt-based electrocatalysts are prepared in organic solutions, probably because the size or shape are more easily controlled than those in aqueous solutions. However, most organic solvents are expensive, toxic, and environmentally unfriendly. Furthermore, reactions in organic solvents usually need to occur at higher temperatures, which increases the production cost of Pt-based electrocatalysts and makes it more difficult to maintain a uniform reaction temperature in a scaled-up reaction system. Organic solvent molecules tend to strongly adsorb on the surface of Pt-based electrocatalysts and carbon, which makes postsynthesis treatment more challenging. Taking economic and environmental considerations into account, aqueous reaction systems may be more suitable for mass production, and future experimental research should be focused on how to effectively control the size and shape of Pt-based electrocatalysts in aqueous solutions.

### Postsynthesis Treatment

Since ligands, referring to those that can adsorb on or even bind to the surface of Pt-based electrocatalysts to control their size and shape, are unavoidable in various solution-based methods, ligand issues should be given more attention in regard to electrocatalytic applications. It has been found that not only is the synthesis process crucial for the performance of the final Pt-based electrocatalysts, but postsynthesis treatments also have an important influence. Therefore, simple, inexpensive, environmentally friendly, reliable, and controlled postsynthesis treatments need to be developed, and during the synthesis process, the amount of ligand used should be minimized or ligands that can be easily removed afterward should be adopted.

Taking the above challenges into consideration, the size and shape of carbon-supported Pt-based electrocatalysts in aqueous systems are suggested to be effectively controlled by (1) using functionalized carbon supports instead of pristine carbon, (2) modifying the pH, reaction temperature, or reduction capability of reductants to control nucleation and growth, (3) using some structure-directing agents with small molecules, e.g., halide ions and CO, which are easy to remove, and (4) selecting structure-directing agents with the same terminal group of –SO_3_H as perflorosulfonate acid ionomers, e.g., sodium dodecyl sulfonate, the residues of which on electrocatalyst surfaces can probably contribute to proton transport in PEMFCs.

## Concluding Remarks

In this review, recent progress related to the controlled synthesis of carbon-supported Pt-based electrocatalysts for PEMFCs has been examined, including the functionalization of commercial carbon supports, methods for loading Pt-based electrocatalysts onto carbon supports, synthesis of spherical and nonspherical Pt-based electrocatalysts, and postsynthesis treatments.

Carbon acts not only as a physical support with electronic conductivity but also provides structural and surface properties that impact the activity and durability of supported Pt-based electrocatalysts. The surface properties of commercial carbon materials are typically modified by surface functionalization, e.g., by the introduction of oxygen-containing groups, heteroatom doping (N, S, or B), the introduction of functional groups or groups, molecules, or polymers with functional groups, and the introduction of metal oxides. The size and dispersion of Pt-based electrocatalysts on functionalized carbon are better controlled due to the increased number of nucleation sites or anchoring sites. The metal–support interaction is also increased via the surface functionalization of carbon; thus, supported Pt-based electrocatalysts commonly show enhanced activity and durability.

There are two approaches commonly used to load Pt-based electrocatalysts onto carbon supports, i.e., one-pot synthesis and ex situ mixing methods. From a commercial perspective, one-pot synthesis methods are desired to prepare carbon-supported Pt-based electrocatalysts to simplify the production process and hence the cost of catalyst fabrication. However, for easy control of desirable shapes and uniform sizes, ex situ mixing methods will be more suitable for Pt-based electrocatalysts because the addition of carbon is commonly disadvantageous to controlling the shape and size of Pt nanoparticles. Therefore, previous studies in the literature have mostly used ex situ mixing methods, which are multistep production processes, thereby increasing the space and equipment needed for production and leading to a higher production cost.

Spherical Pt-based electrocatalysts are easy to prepare and hence are the common shape in practice. However, nonspherical Pt-based electrocatalysts usually have better performance and durability and have been synthesized successfully by appropriately controlling of nucleation and growth, resulting in Pt nanostructures with a controlled size and shape, e.g., polyhedrons, nanocages, nanoframes and 1D or 2D nanostructures. The synthesis process can be carried out by properly selecting the used materials, designing the reaction system, and controlling the reaction conditions. Compared with the synthesis of spherical Pt-based electrocatalysts, the reaction systems for nonspherical Pt-based electrocatalysts are commonly complex, and the reaction conditions are demanding. Therefore, even though nonspherical Pt-based electrocatalysts generally have higher activity and durability, there is still a lack of commercial nonspherical Pt-based electrocatalysts.

The prepared Pt-based electrocatalysts commonly need postsynthesis treatment to remove contaminants adsorbed on their surface. Simply washing with a copious amount of solvent is usually not sufficient to clean strongly adsorbed organic ligands in most cases, and further purification is needed. Further purification methods include acid washing, thermal annealing, UV-ozone irradiation, and electrochemical cleaning. However, most of these purification processes are possibly detrimental to the performance of the final Pt-based electrocatalysts.

Finally, perspectives on the challenges and future directions are outlined, including the development of new methods to maximize the potential advantages of functionalized commercial carbon supports and developing new reaction systems to increase the yield of carbon-supported Pt-based electrocatalysts in one batch, especially nonspherical carbon-supported Pt-based electrocatalysts. Organic reaction systems and organic ligands should be avoided whenever possible to simplify reaction systems and postsynthesis treatments, which will also contribute to low-cost fabrication and environmental protection.
